# Psychotherapy in pain management: New viewpoints and treatment targets based on a brain theory

**DOI:** 10.3934/Neuroscience.2020013

**Published:** 2020-07-14

**Authors:** Robert A. Moss

**Affiliations:** North Mississippi Regional Pain Consultants, 4381 Eason Blvd., Tupelo, MS 38801 USA

**Keywords:** chronic pain, brain, psychotherapy, cortical column, Dimensional Systems Model, Clinical Biopsychological Model, personality

## Abstract

The current paper provides an explanation of neurophysiological pain processing based the Dimensional Systems Model (DSM), a theory of higher cortical functions in which the cortical column is considered the binary digit for all cortical functions. Within the discussion, novel views on the roles of the basal ganglia, cerebellum, and cingulate cortex are presented. Additionally, an applied Clinical Biopsychological Model (CBM) based on the DSM will be discussed as related to psychological treatment with chronic pain patients. Three specific areas that have not been adequately addressed in the psychological treatment of chronic pain patients will be discussed based on the CBM. The treatment approaches have been effectively used in a clinical setting. Conclusions focus on a call for researchers and clinicians to fully evaluate the value of both the DSM and CBM.

## 1. Introduction

The opioid crisis is a frequent topic covered in the news media. Rather sweeping changes are occurring, including stricter laws on the use of pain medications in isolation and with other medications, such as benzodiazepines. The growing number of lawsuits and disbanding of the American Pain Society are examples of the fallout of the growing public awareness of opioid addiction and new governmental regulations. Due to the fact that many patients treated with opioids are already receiving antidepressant medications and other psychotropic drugs, there is a need for additional treatment approaches with an opportunity for psychology to make significant contributions in the pain management arena.

In support of the need for involvement of psychological treatment in chronic musculoskeletal pain cases, Tseli et al. [1] performed a systematic review of prognostic factors for long term physical functioning following multidisciplinary rehabilitation. They found that pain chronicity and intensity did not predict physical functioning following multidisciplinary treatment. Instead, better outcome was predicted by low levels of pre-treatment emotional distress and low levels of cognitive and behavioral risk factors, as well as high levels of protective cognitive and behavioral factors. The authors suggested that treatment targeting these factors “may perhaps provide an opening for yet untapped clinical gains.” (p. 148)

In 1977, Engel [2] proposed a new conceptual model of illness that included social, psychological, and behavioral dimensions. The biopsychosocial approach [3] is commonly used to both conceptualize and guide treatment in psychotherapy with pain disorders. Meints & Edwards [4] explained “the biopsychosocial approach describes pain and disability as a multidimensional, dynamic interaction among physiological, psychological, and social factors that reciprocally influence one another, resulting in chronic and complex pain syndromes.” (p. 169)

Gatchel, McGeary, McGeary and Lippe [5] discussed the importance of interdisciplinary chronic pain treatment, with involvement of physicians, nurses, psychologists, physical therapists, and occupational therapists. In his book on pain management, Gatchel [6] discussed a comprehensive approach to treatment of chronic pain patients using a biopsychosocial perspective with an emphasis on psychological approaches to treatment. He suggested the possible use of group therapy and marital/relationship counseling due to the importance of a patient's social support. In relation to individual psychotherapy, he discussed the use of relaxation/biofeedback, attention/distraction, cognitive-behavioral interventions, assertiveness/social skills, increasing reinforcing activities, life planning, managing secondary gains, secondary loss issues, and motivational enhancement. Little has changed from the overall program he discussed in 2005 [5].

In a detailed review of psychosocial factors contributing to chronic pain outcomes, Meints and Edwards [4] provided information that can serve to outline the potential targets of psychological treatment. In relation to affective factors, it is noted that pre-morbid psychological dysfunction is a risk factor for the development of chronic pain symptoms. They commented that this interpretation contrasts to the frequent one that psychological symptoms are a consequence of chronic pain. This is one factor leading to the recommendation for early interdisciplinary intervention for at risk populations in the prevention of disabling chronic pain conditions [7].

The next area discussed by Meints and Edwards [4] is trauma. They noted there are strong prospective links between early-life trauma (i.e., physical, psychological, and sexual abuse) and the later development of chronic pain. Additionally, they discuss the evidence that later posttraumatic stress disorder (PTSD) amplifies the predictive effects of childhood abuse on chronic pain, making the observation of just how entrenched and enduring are the damaging effects of such abuse. In their literature review, Burke, Finn, McGuire, and Roche [8] indicate there is evidence of alterations in some neurobiological substrates (e.g., hypothalamic-pituitary-adrenal axis, neurotransmitter systems) as a result early-life stress that are correlated with chronic pain conditions. Similarly, Meints and Edwards [4] discuss the correlational date on brain cortical and subcortical areas involved with chronic pain. However, to the current author's knowledge, there has been no theoretical discussion involving brain mechanisms on the possible manner early-life trauma or other psychological factors contribute to chronic pain. In total, it appears that early-life trauma is seen as a static mechanism used primarily as a risk factor as opposed to being considered a dynamic, but ongoing and persistent, mechanism that may be modifiable with appropriate psychological treatment. This will later be discussed in more detail. Examples of clearly static and non-modifiable psychosocial mechanisms related to chronic pain are gender and race that Meints and Edwards [4] also discuss.

Another area covered by Meints and Edwards [4] involves social/interpersonal factors. The studies reviewed focused on either non-pain-related global social support or pain-related social responses. They note it is clear that interactions between chronic pain patients and their significant others can both facilitate and impair adjustment. Such factors as a partner's depressive symptoms or avoidant and anxious attachment styles are associated with increased problems related to pain. Patients with anxious or insecure attachment styles have been shown to be at elevated risk for poorer outcomes in pain treatment. In relation to the work environment, the authors note the importance of social support and satisfaction with co-workers as predictors of pain-related disability. It appears that the main proposed manner in which the social area has been addressed involves couple's interventions interpersonally and via occupational or vocational counseling related to the work situation. Thus, there is a lack of discussion of how important social and work relationships can be addressed in individual psychotherapy despite the role social relationships play in chronic pain syndromes. Sturgeon and Zatura [9] suggested more explicit focus on addressing interpersonal distress and enriching one's relationships are underexplored areas of chronic pain treatment. In relation to interpersonal factors in individual psychotherapy, Meints and Edwards [4] note the importance of establishing a sound therapeutic relationship, although there are only a few related studies to date.

The current paper presents a brain-based model with the potential for explaining brain mechanisms involved with chronic pain and a discussion of psychological treatment targets that have received little to no attention. There will first be a brief discussion of cortical pain perception based on previous research. This will be followed by an explanation of a brain model based on cerebral cortical columns and how this relates to the prior research. Based on the applied Clinical Biopsychological Model (CBM), there is a discussion related to identifying and treating influential negative emotional memories with the goal of reducing the psychological and physical impact of those memories. There is next an explanation of the two meta-traits (i.e., plasticity and stability) of the Five Factor Theory of personality based on the CBM and how the new viewpoints translate into specific recommendations on the most effective ways for pain patients to behaviorally interact with those in their social network.

## 2. Pain perception and the brain

There are a number of body locations and syndromes that involve chronic pain, which is generally viewed as pain lasting longer than three to six months. The current paper will not attempt to discuss each pain condition individually and is not intended to be a critical review. There is some evidence that there may be gender differences in brain alterations associated with chronic pain [10], but that will not be addressed as that aspect awaits future studies prior to drawing any meaningful conclusions. Additionally, there will not be a detailed discussion of how studies involving induced pain, often in pain-free subjects, may differ from chronic pain patients in relation to involved brain areas. However, in their meta-analysis of 266 cutaneous pain fMRI studies, Tanasescu, et al. [11] found that chronic pain and non-pain controls have a shared neural pain signature with the only exception being when the most painful body part is stimulated with chronic pain patients.

There will be a discussion of brain areas as related to transition from acute pain to a chronic pain due to its potential importance in relation to psychological treatments. Interestingly, some studies involving chronic pain patients, as opposed to pain-free subjects, report possible differences in behavioral effects. For example, Jegindo, et al. [12] found prayer to reduce significantly both pain intensity and pain unpleasantness from electrical stimulation in pain-free subjects. In contrast, chronic pain patients who used prayer and hope as a coping strategy reported greater disability in a different study [13].

The ensuing discussion of the brain areas involved with chronic and acute pain is presented to provide information on the areas that have been associated with pain perception and control with the goal of next explaining how the cortical column theory explains the involvement of each area. In their review of 27 studies involving chronic low back pain patients, Kregel et al. [14] concluded there is moderate evidence for regional changes in gray and white matter in addition to altered functional connectivity. Seminowicz and Moayedi [15] indicted there is some level of convergence across different chronic pain disorders. As an example, they mention studies that have shown decreased gray matter volume in the dorsolateral prefrontal cortex (dlPFC) in patients with chronic low back pain, migraine, trigeminal neuralgia, hypnic headache, chronic post-traumatic headache, hip osteoarthritis, and complex regional pain syndrome. In an earlier review of several chronic pain conditions, May [16] noted overlap across pain conditions related to changes in the cingulate cortex, orbitofrontal cortex (OFC), insula, and dorsal pons. May concluded that the gray matter change observed in chronic pain patients may be the consequence of frequent nociceptive input and, if so, should be reversible when pain is adequately treated.

In support of May's [16] conclusion, a study was done by Rodriguez-Raecke, et al. [17] when comparing brain MRI in hip arthritis patients in a pre-surgery pain state to a post-surgery pain free state. Compared to controls, patients with chronic pain had significantly less gray matter in the anterior cingulate cortex (ACC), insular cortex and operculum, dlPFC, and OFC. When pain free after recovery, a gray matter increase occurred in many of the same areas. The authors concluded that gray matter abnormalities in chronic pain are not the cause, but secondary to, the disease.

## 3. Pain neurophysiology

**Figure 1.**
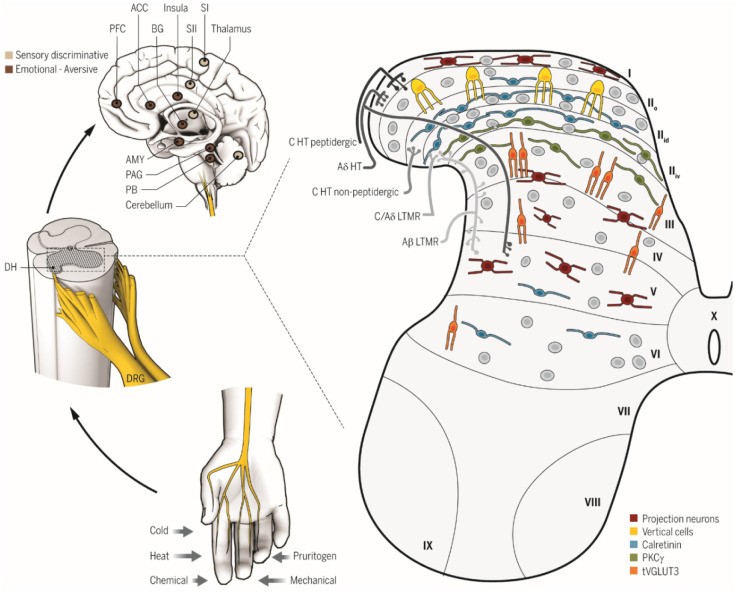
Overall organization of somatosensory circuits. Cutaneous sensory neurons (DRG) are activated by a variety of stimuli (bottom left) and project to the spinal cord dorsal horn (DH, middle left). In the DH (right), the central terminals of high-threshold nociceptors (HT) are located in the most superficial laminae [lamina I to the dorsal part of lamina II (II_id_ )] and lamina V. Low-threshold mechanoreceptors (LTMR) preferentially end in the deep dorsal horn [ventral part of inner lamina II (II_iv_) to lamina V]. The spinal cord is divided into 10 laminae (the DH is I to VI) and is composed of numerous neuronal populations. Some identified populations are organized in longitudinal layers (only excitatory neurons are represented): neurons transiently expressing VGLUT3 (tVGLUT3, orange) in laminae III and IV, PKC_γ_ (green) in lamina II_iv_, calretinin (blue) in outer lamina II (II_o_) and lamina II_id_, vertical cells (yellow) in lamina II_o_, and projection neurons (red) in laminae I, IV, and V. Projection neurons send information to the brainstem and thalamus and then on to several brain regions implicated in sensory-discriminative (upper left, light brown) and emotional (upper left, dark brown) sensory perception. ACC, anterior cingulate cortex; SI (II), primary (secondary) somatosensory cortex; PAG, periaqueductal gray area; PB, parabrachial nucleus; AMY, amygdala; PFC, prefrontal cortex; BG, basal ganglia [Reprinted from Piers and Seal (2016) by permission of authors and the American Association for the Advancement of Science].

As shown in Figure 1, nociceptors convey information to the spinal cord along the Aδ (faster conducting, relatively larger, and myelinated axons) and C (slower conducting, small, and unmyelinated axons) fiber pathways [18]. Most nociceptive afferents make synaptic contact in Rexed laminae I and II of the spinal cord, while low threshold Aβ afferents (mainly sensitive to touch, but also second-order nociception based on Hendry and Hsiao [19]) synapse in laminae III to V [20]. There are two brain areas receiving direct input from the C fibers; the parabrachial nucleus (PBN) and the thalamus [20]. The PBN sends input to the central nucleus of the amygdala (CeA) involved in emotional reactions to pain [21] which is discussed in more detail later in this section. The laminae IV and V input goes to the thalamus and periaqueductal gray (PAG). The PAG activation can allow pain control via activation of the descending serotoninergic and adrenergic neurons which, in turn, drive laminae II interneurons [19]. These interneurons release enkephalins onto incoming nociceptor afferents and spinothalamic neurons.

Via the spinothalamic tract there is nociceptor input to the thalamus that involves two separate cerebral cortical pathways. There appears to be agreement on the path involved with the discriminative, localized component of pain (often referred to as first pain) involving the larger myelinated axons input to the spinal cord [19]. Convergence of lamina I nociceptive input and laminae IV and V touch input occurs in the ventral posterior complex (VPC), which includes the ventral posterolateral (VPL), ventral posteromedial (VPM), ventroposterior inferior (VPI) nuclei, and intralaminar nuclei (ILN). Each of the VPC nuclei project to S1, with VPI also projecting to S2. Of great relevance to the psychological impact of chronic pain is the medial pain pathway to the cortex. There are thalamic connections from the smaller, unmyelinated peripheral C fibers associated with the punishing, motivational, and emotional aspects of pain (often referred to as second pain). This involves projections including and beyond the VPC which includes the ventrocaudal mediodorsal nucleus (Mdvc), medial nucleus of the posterior group (POm), and posterior part of the ventral medial nucleus (VMpo). Mdvc provides input to the ACC while POm and VMpo provide input to the insula (Ins). Vogt (2005) notes both spinothalamic tract and parabrachial nucleus (PBN) input to the ILN which provides input to the middle cingulate cortex (MCC).

The “pain matrix” [22] refers to the network of brain regions involved with the sensory-discriminative and cognitive-affective aspects of pain. In reaction to noxious stimuli, Peirs and Seal [18] note fMRI has shown coordinated activation of the thalamus, ACC, insular cortex, S1 and S2 somatosensory cortices, prefrontal cortex (PFC), basal ganglia, cerebellum, and amygdala. Figure 1 depicts those areas of involvement. However, those authors also note that many of these same areas can be activated by non-nociceptive stimuli. This latter observation leads to a very important point.

The position of the current paper is that different circuits of cortical columns are contained within the same brain regions and it is necessary to understand the design to better interpret the role of the involved circuitry. This is analogous to circuit boards in a television; a board involved with sound may interconnect with the one involved with video, and both activate when the television is switched on. However, it is impossible to know how the boards function and fix problems without a knowledge of the individual circuits of each board. This example also provides an understanding that there are circuits within the boards and a circuit connecting the two boards, just as the current paper discusses interareal cortical circuits of columns and the connection of those localized columnar circuits to other cortical areas and subcortical structures. The typical level of discussion in brain scan studies involves the areas of the cortex, which is the same as discussing results at the level of the circuit boards. The viewpoint of the current paper is supported in a recent article describing a large-scale dynamical circuit model of the human cortex [23]. Their findings highlight the importance of local properties and associated hierarchical specialization on the large-scale organization of human cortical dynamics. In relation to physical pain versus emotional pain (i.e., social rejection), Woo et al. [24] provided evidence that there are dissociable fMRI patterns that imply the neuron-level population codes are different despite these often activating the same areas, such as dorsal ACC (dACC) and anterior insula (aIns).

Wiech [25] discussed the influences of cognitive processes on pain perception in relation to neural structures. Factors documented to impact pain perception are attention, anticipation, catastrophizing, reappraisal, and perceived control. Meints and Edwards [4] provide a detailed discussion of the research done on pain-specific psychosocial constructs to which the interested reader is referred. They note active problem-solving coping strategies are associated with positive affect, better psychological adjustment, and decreased depression in contrast to passive strategies which have been linked to poorer outcomes. Catastrophizing is linked to higher pain levels and, like praying and hoping, is associated with increased interference from pain. Positive outcome expectancy is related to increased feelings of control, use of active coping strategies, and better functional performance. They note that expectations play a key role in placebo analgesia. Reinterpreting pain sensations has been shown to lower pain ratings, but typically to have weak correlations with pain interference and physical disability. Similarly, ignoring pain has generally been shown as a poor predictor of pain interference, disability, and pain severity. Thus, an accurate theory of cortical functioning must be able to provide an explanation of such findings.

Reddan and Wager [26] discuss the use of multivariate predictive modeling, called the Neurologic Pain Signature (NPS), in an attempt to better define brain features related to pain. They report that greater activity in the ACC, Ins, S2, and thalamus, predicted more pain. In contrast, greater activity in the ventromedial PFC (vmPFC) and precuneus indicate less predicted pain. Wiech [25] notes the “pain matrix” has historically been divided into the sensory-discriminative system, which includes the lateral thalamus, S1, and S2, and the cognitive-affective system, which includes the aIns and ACC. Thus, the NPS includes locations of both divisions. Wiech further discusses the descending pain control system that includes the dlPFC, rostral ACC, and PAG. As previously mentioned, the descending pathway activation and functional connectivity has been associated with reported pain relief. In relation to dlPFC, Seminowicz and Moayedi [15] discuss several studies that showed reversal of gray matter volume loss with successful treatment or symptom resolution, supporting the structural plasticity of areas showing volumetric changes. In their meta-analytic review, Tatu et al. [27] found the gray matter alterations are not random and resemble the pattern of functional connectivity of areas involved with chronic pain processing. That review also found lateralization of volumetric changes and connectivity such that there is greater right-sided involvement.

Woo et al. [28] discussed brain locations beyond the NPS which primarily involve areas in response to noxious stimulus-intensity encoding. In addition to the areas responding to painful stimuli, there are other areas involved with pain modulating effects of psychological interventions (e.g., placebo treatment, cognitive self-regulation, and perceived control). The regions showing positive (increased) pain-predictive weights are dorsomedial PFC (dmPFC), middle temporal gyrus, caudate, and ventrolateral PFC (vlPFC). The regions showing negative (decreased) pain-predictive weights are the vmPFC, nucleus accumbens (NAc), parahippocampal cortex (PHC), and posterior dlPFC. The authors noted studies show there are differences across studies in relation to effects on pain. For example, vmPFC activation is often associated with reduced pain in healthy controls, but associated with pain catastrophizing and increased pain in chronic pain patients. This supports the need to consider group differences and cognitive context when considering the role of each area.

Woo, Roy, Buhle, and Wager [29] found NAc to vmPFC connectivity mediates the effects of cognitive self-regulation on pain independent of noxious stimulus intensity. Wiech [25] proffered the idea that the mesolimbic network may integrate sensory, cognitive, and affective aspects to give rise to the unified pain experience. The role of attention on pain perception was also discussed by Wiech, noting the aforementioned descending pain control system as one circuit. One of the other two systems is the “salience network” that is involved in the detection of biologically relevant stimuli. It involves dlPFC, MCC, aIns, and temporoparietal junction (TPJ). The other attention system is the default mode network (DMN) that includes the mPFC, posterior cingulate cortex (PCC), precuneus, lateral parietal lobe, and medial temporal lobe. The DMN is engaged when focused away from pain while the salience network activates when attention is spontaneously focused on pain. In that regard, it has been shown [30] that both chronic pain and non-pain subjects show similar alterations in DMN function during acute noxious stimulation. The authors concluded that was likely that the presence of pain, and not its chronicity, that accounts for attentional process alterations.

A final system of importance was identified in one study [21] and includes the amygdala. The PBN receives collateral projections (i.e., the main projections are to the thalamus as previously discussed) from peripheral nociceptors [21],[31]. The Cai et al. [21] study involving rats found that activation of the PBN relays the peripheral pain signals to CeA and is sufficient to cause negative emotion behaviors reflective of depression, anxiety, and aversion. This suggests that peripheral pain signals can directly activate emotional responses without cortical processing. In contrast, activation of the excitatory pathway from basolateral amygdala (BLA) opposes the negative emotion behaviors and induces behaviors of reward. The BLA conveys processed corticolimbic signals to CeA. The authors concluded the BLA to CeA circuit may be a top-down mechanism for cognitive control of negative emotions related to pain. In their discussion of the PBN as a hub for pain and aversion, Chiang at al. [32] discussed the research supporting the PBN as connecting the ascending pain pathways with descending pain-modulation pathways. Lateral PBN (lPBN) projects to the PAG and its stimulation produces analgesia. In contrast, lPBN output to the rostral ventromedial medulla has a net pronociceptive effect that is pronounced in persistent pain states.

In summary, the following areas have been identified in brain pain perception and control: thalamus, S1, S2, Ins, middle temporal gyrus, precuneus, PHG, dlPFC, vlPFC, vmPFC, OFC, ACC, MCC, NAc, caudate, putamen, PAG, PBN, amygdala, and cerebellum. The salience network and DMN have been implicated in attention toward or away from pain. In relation to neuroimaging in animal models, chronic pain brain alterations are most commonly observed in regions associated with emotion and motivation, including PFC, ACC, hippocampus, amygdala, basal ganglia, and NAc [33]. The discussion will now shift to the columnar brain model.

## 4. Dimensional-Systems Model (DSM)

Consistent with Luria's [34] views, the DSM [35] considers broad brain areas as influencing higher cortical functions. There were five systems originally proposed. The sensory input system focuses primarily on tactile, auditory, and visual input as being the most influential in higher functions and related to the manner by which processing occurs in specific cortical areas. The arousal system involves the power supply to the cortex necessary for processing and memory storage which can be selectively enhanced based upon ongoing biological needs and emotions. The attention–memory system involves the structures and mechanisms by which incoming sensory information is selected and subsequently stored in memory at the cortical level. The cortical system involves the means by which the columns interact to provide processing, analyses, and responses. Finally, the motor system describes the output level of the system by which environmental manipulations occur. Of key relevance to the clinical applications of the model is that all memory storage of information relevant to psychotherapy occurs at the cortical level and increased emotional arousal leads to enhanced memory storage. The current discussion will focus primarily on the cerebral cortical system and its involvement with those areas identified as associated with chronic pain.

The cortical column has been proposed as the binary digit (bit) for all cerebral cortical processing [35]. This simply means that each cortical column represents specific and discrete information. Based on the assumption that circuits of columns are the manner that all higher cortical functions occur, then the role of all other involved brain structures outside the cerebral cortices is to facilitate columnar circuitry. For example, I proposed the single purpose of the hippocampus is to allow the binding of parallel circuits of columns [36]. In the current paper, I will expound upon the proposal [37] that the basal ganglia provide the inhibition for all cortical column circuits. I also propose that the cingulate cortex provides the selection and implementation (volitional control) for all voluntary, as opposed to automatic, actions. In relation to automatic behaviors, I later explain my proposal that the role of the cerebellum is to allow activation of columnar circuits associated with overlearned actions (e.g., habits) without cortical attention involvement. If accurate, this means that the observed activation of the cerebellum in novel tasks is not one of aiding in immediate processing and actions; instead, it is brought online in case the novel actions become repeated frequently so that it can eventually directly activate the lower-order action columnar circuits associated with the behaviors based on specific sensory input without frontal lobe attention mechanisms.

Briefly, a column is comprised of several hundred minicolumns each of which may contain approximately 100 to 200 neurons [38]. The diameter may vary from approximately 0.4 to 1.0 mm, depending upon the cortical location and species. Moss [35] suggested that columns overlap such that some minicolumns are shared. Moss et al. [39] added the propositions that only the outer minicolumns comprised the actual columnar bit and that minicolumns may also overlap. Although other authors [40] suggested the cortical column likely has no functional significance, Moss & Moss [41] addressed the concerns raised by Horton & Adams [40] based on the theoretical discussion of the manner in which columns may dynamically form to accommodate different sensory inputs (e.g., various languages) and associated actions.

Recent high-resolution fMRI with humans has shown columnar-like structures in V1 [42],[43], V2 and V3 [44], V3a [45], hMT [46], and ventral temporal cortex showing differential representations of categories and domains [47]. Of particular relevance is the recent study by Schneider et al. [48] in which high resolution fMRI provided proof that in the human middle temporal visual complex, columnar clusters meet the criteria of content-specific neural correlates of consciousness (NCC). NCC refers to the minimal set of neuronal mechanisms jointly sufficient for any one conscious percept [49]. In the Schnieder et al. [48] study, the perceived axis of motion (i.e., horizontal versus vertical) was associated with distinct columnar clusters which supports the viewpoint of the column as the cortical bit.

To assist in understanding cortical processing based on the DSM, two points will be helpful. First, everything works in circuits. Second, the components in the circuit determine what functions occur. For example, a simple electrical circuit involves a power supply, a component or device (e.g., light bulb, buzzer), and the wires connecting these. Usually there is a switch that serves to activate and deactivate the circuit. If a light bulb is in the circuit, it glows when the switch is in the on position. If there is a separate circuit with a buzzer, the buzzer does not activate when the light bulb circuit's switch is on because it is not connected to that switch. If both the buzzer and light bulb are in the same circuit (i.e., in “series” or serial processing) then both light and sound result with switch activation. Similarly, if the separate light bulb and buzzer circuits are connected to the same switch (in “parallel” or parallel processing), both light and sound occur when switched on. Therefore, it is easy to see that if components or devices are not connected to a given circuit then there is no expectation they will activate.

Moss et al. [39] provided a physiological definition of memory which involves the strengthening of synaptic connections in any given circuit of cortical columns that are used in information processing. Forgetting is the result of weakened synaptic connections which means the downstream columns in the circuit fail to activate. In this case, the column's activation by one or more other columns fails to be maintained. However, with structural changes, such as axonal sprouting and increased dendritic spines between neurons of columns, then the likelihood of “forgetting” is greatly reduced because the connections are resistant to disruption and damage.

There are several important aspects tied to the definition. First, all memory tied to higher functions occurs at the cortical level and all memory involves the same mechanisms. The perceived quality of a memory is a function of the information represented by the columns involved, in the same manner that the previously discussed light bulb and buzzer determine the output of simple electrical circuits. Thus, columns in the temporal cortex that code for spoken words are perceived as spoken words when activated, while those in the parietal lobe that code for body sensations are perceived as sensations when activated. Similarly, explicit (i.e., declarative) memory and implicit memory are both circuits of columns, with the qualitative distinction being whether the “interpreter” [50] has direct access to the memory. As will be discussed, for the majority of humans the left (as opposed to the right) lateral inferior frontal area is the theorized location of self-talk, or internal verbal dialogue, and is what defines declarative memory (i.e., being able to verbally explain what is being remembered) and which also allows verbal schemas [37],[51]–[54].

The detailed explanations of the cortical neurophysiology and supporting research are available in a series of articles to which the interested reader is referred [35],[36],[39],[41]. The involvement of the basal ganglia and cerebellum as related to the columnar model have not been previously discussed in detail and some of the relevant research will be discussed in a little more detail in the current paper. The applied CBM has also been discussed in detail related to the DSM [37],[53]–[56] and only certain aspects will be discussed in the current paper.

## 5. Dimensions of the DSM

The dimensions of cortical column processing are described in Table 1 and these explain the both the modes and locations of cortical processing. Lateralization of cortical functions is explained on the basis of the number of columns from the point of sensory input to the point of response output. The right hemisphere has relatively fewer columns in circuits compared to the left. The result is that the left hemisphere handles more detailed processing and is relatively slower in processing speed compared to the right. Notably, each side can simultaneously process the same sensory information and contribute its part to interhemispheric responses. An example has to do with emotional processing and responses. All brain structures, with the exception of the pineal body, are paired. This means that each cortical hemisphere has its own subcortical structures related to emotions (e.g., amygdala, NAc). This supports the conclusion that both cortices can be involved in both positive and negative emotions. For example, negative emotional reactions to the verbal aggression of one's boss or spouse can result simultaneously from words processed in the left hemisphere and facial expressions and voice intonations processed in the right hemisphere. This concept will be used to explain how some forms of psychological treatment may adequately address one hemisphere's involvement while failing to impact the other hemisphere's circuits.

**Table 1.** Dimensions of Cortical Column Organization in the Dimensional Systems Model. Reprinted from Moss & Moss [41].

Dimension Name	Description of Dimension
Internal-external	The medial cortical columns code stimulus information that is internal and self-referential while the lateral cortex codes for external stimuli. Intermediate or transitional zones code for combinations of both.
Proximal-distal	In relation to proximal versus distal to the body stimulus coding, the central sulcus is considered the most proximal cortical location. The post-central sulcus parietal cortical area would code for somatosensory (i.e., body sensation) stimuli. Both vision (occipital lobe) and audition (temporal lobe) involve distal sensory information. The pre-central sulcus primary motor strip involves the body directly while anterior prefrontal processing involves information manipulation largely independent of the body.
Simultaneous-sequential	Ventral cortex processes in a sequential manner and dorsal cortex in a simultaneous manner, with intermediate areas using both modes of processing.
Reception-action	The parietal, temporal, and occipital lobes contain all receptive, or sensory, information while the frontal lobes code for all action-related information.
Unorganized-organized	Receptive information progresses from less-organized, or lower-order, information to more-organized, or higher-order, information (i.e., coding) as the stream moves away from the primary sensory receiving areas (i.e., bottom-up processing). On the other hand, the frontal action columns progress in a rostral to caudal more-organized, or higher-order information to less-organized, or lower order information (i.e., decoding) as the stream goes toward the premotor and primary motor areas. The frontal action columns' control of posterior lobe receptive columns is also present (i.e., top-down processing).
Analytical-Global	Each cortical hemisphere acts as a separate, albeit interconnected, processing unit which means that each of the aforementioned dimensions is contained within each hemisphere. However, there are fewer columns from the time of sensory input to the response level in the right hemisphere. This means that the right cortex can process information faster, but with fewer details (i.e., global processing). The greater number of interconnected columns in the left hemisphere allows more detailed processing and memory storage (i.e., analytical processing)

The other dimensions exist in both hemispheres. The unorganized to organized dimension involves how the sensory information is coded. In contrast to the often-stated view that information from the primary receiving areas for the senses is “extracted” to allow higher functions, the DSM indicates it is summed. Primary sensory information is received at the cortical level in the exact manner it is received by the sensory organs (e.g., tonotopic columns in the temporal lobe, retinotopic in the occipital lobe). For example, auditory columns are activated for specific sound frequencies and, when activated, convey their information outward. The location where the activated sound frequency columns' efference cross becomes entrained as a new higher-order column that represents a combination of its associated lower-order columns. The higher-order columns then send along their information and entrain additional higher-order columns. In relation to speech processing, phonemic and syllabic columns form, based on the sound frequency columns, and their output form word columns. In the frontal lobes the coding process is reversed such that rostral higher-order columns activate the caudal lower-order columns. The lowest-order columns are the ones involved in controlling the response output, such as in the primary motor cortex for mouth and tongue movements when speaking. In relation to higher order association cortices connectivity, it has been shown [57] that intra-regional connectivity for the frontal, temporal, and parietal lobes is dense and generally graded which fits with the foregoing proposal. Jung et al. [58] provided support that there is a direct relationship between the connectivity of tertiary association cortical areas identified in task-free data-sets and higher cognitive functions (i.e., perception, cognitive control, language, visuospatial function, and memory).

The next dimension involves reception and action. The posterior cortical areas (occipital, parietal, and temporal) involve incoming information while the frontal lobes control actions. The DSM proposes that for each posterior column, an associated action column will form. Each higher-order frontal action column has control over its associated higher-order receptive column, as well as control over the lower-order action columns that form based on the lower-order receptive columns feeding into the higher-order receptive column. In the previously discussed case of speech, a receptive word column in the temporal lobe is connected to its associated action word column in the frontal lobe. The phonemic and syllabic columns that created the receptive word column have their respective action columns in the frontal lobe. The frontal lobe action word column can activate the syllabic and phonemic action columns in the frontal lobe if the word is to be spoken, or it can activate its associated receptive word column only as in the case of inner speech. This appears consistent with Jung et al.'s [57] finding that inter-lobe connectivity is relatively discrete and regionally specific such that only small sub-regions had long-range connections to another lobe.

Another dimension involves internal versus external stimuli processing. The lateral cortices code for information from auditory, visual, and somatosensory input, all of which involve information arriving from the external environment. The medial cortex codes for information involving internal and self-referential stimuli. An example is the medial frontal lobe being involved with a voluntary decision to initiate action (internal) and the lateral frontal cortex with the behavioral response (external). The convergence zones (e.g., insula, frontal pole, supplementary motor area) code for the combination of internal and external information involving serial circuits, while other medial areas (e.g., retrosplenial cortex, medial temporal lobe cortex) code for parallel cortical circuits involving a combination of internal and external processing (e.g., simultaneous combination of egocentric and allocentric spatial information during navigation). The action columns for the combined internal and external information will be located in the medial frontal lobe (e.g., medial temporal lobe cortex connects to medial frontal lobe cortex).

The simultaneous versus sequential processing refers to the dorsal versus ventral locations. Somatosensory information arriving in the parietal lobe may involve processing information at multiple body locations at the same time and the simultaneous processing mode appears to characterize the dorsal portion of the parietal lobes. In contrast, auditory information involves processing of information arriving sequentially and this characterizes the sequential processing mode of the ventral temporal lobes. Visual processing involves both simultaneous and sequential (e.g., the dorsal “where” and ventral “what” information streams) which can explain its location between the dorsal and ventral cortex. The convergence zones of the parietal, temporal, and occipital lobes (e.g., TPJ) involve a combination of simultaneous and sequential receptive information processing (e.g., motion detection, syntax).

In relation to the dimension of proximal versus distal to the body stimulus coding, the central sulcus is considered the most proximal cortical location. The post-central sulcus parietal cortical area codes for somatosensory (i.e., body sensation) stimuli. Both vision (occipital lobe) and audition (temporal lobe) involve distal sensory information. The pre-central sulcus primary motor strip involves the body directly while anterior prefrontal processing involves information manipulation largely independent of the body. A similar proposal with be discussed in relation to the medially-located cingulate cortex.

### 5.1. Columnar circuitry

The DSM proposes that all information arriving in the posterior cortices involves feedforward processing. This means that visual, auditory, and somatosensory information arriving at the primary receiving areas is immediately passed along, activating higher-order columns. The result is constant processing in multiple circuits in both hemispheres. However, each of the multiple posterior circuits reaches an end point involving its highest-order column. If that receptive column's respective frontal action column is not involved in an ongoing response related to what one is attending, it will not be perceived and simply deactivate. For information to which one is attending, the frontal action columns can reactivate its respective receptive column (typically considered “feedback” or “top down” processing, but in this case the neural signal is actually being sent forward in a circuit sense) and/or activate the associated lower-order columns that can lead to a response output. If the response is being made to an external stimulus, the lateral cortical circuits are always involved, but the medial circuits are involved when one decides to make a response. If the response is being made to an internal stimulus (e.g., hunger, thirst, urge), then medial cortical circuits are involved, but lateral cortical circuits become involved if an external response is required to address the internal stimulus. If all cortical processing involves feedforward circuitry, then two important factors must be explained. First, there must be a way to inhibit the feedforward cortical signals. Second, there must be a way that “automatic” or “habit” behaviors controlled by cortical circuits can occur independent of frontal attention mechanisms. These functions are considered to be the result of basal ganglia and cerebellar involvement, respectively.

### 5.2. Basal ganglia inhibition

The structures of the basal ganglia are the corpus (dorsally located) striatum (i.e., the caudate and the putamen), ventral striatum (NAc and olfactory tubercle), subthalamic nucleus (STN), pallidum (i.e., globus pallidus, GP, and substantia nigra pars reticulata, SN), and ventral pallidum [59]. There are both motor and non-motor loops of the basal ganglia involved with body movement, oculomotor control, prefrontal cognitive functions, and the limbic system. The loops involve the same basic design with input from the cortex to the striatum which then projects to the pallidal structures which in turn project to the thalamus. The thalamus projects back to the cortex. The loops most relevant to psychological treatments are the prefrontal and limbic ones. In relation to the limbic loop, OFC, ACC, and temporal cortices provide input to the ventral striatum, as do the amygdala and hippocampus.

In their integrative review, Hélie et al. [60] provide a discussion of the interaction between the basal ganglia and PFC. They note that, with the exception of V1, the striatum and STN receive input from across the cerebral cortex. The organization of the striatum is relatively simple, with about 95% of neurons being medium spiny (MSN) and the others being aspiny. Excitatory input from between 5,000 to 30,000 cortical pyramidal neurons converge onto a single MSN. Each pyramidal neuron synapses with multiple (estimated to be 10 to 100) MSNs. Focused and convergent cortico-striatal projections are both topographically [61],[62] and functionally [63] organized. There is some divergence of diffuse projections between distal striatal targets which Haber [64] suggested may be a means by which information from functionally distinct cortical regions may be integrated. The described pattern seems compatible with that expected based on the DSM proposed dynamic cortical column formation [41]. In that case, as each new column forms, its minicolumns' pyramidal neurons project to the striatal MSNs, as do all other columns in its circuit. With the reactivation of each column, the tonic inhibition from the GP onto thalamic nuclei must be released prior to the thalamus sending its excitatory signals that allows output from each of the columns in the circuit.

Another aspect discussed by Hélie et al. [60] relates to reinforcement learning. In instrumental learning, dopamine is released into associated synapses to facilitate strengthening, but excess dopamine needs to be removed efficiently to avoid the strengthening of synapses associated with any erroneous response that might next occur. Striatal dopamine is quickly cleared by dopamine active transporter (DAT), while cortical dopamine release changes slowly due to low DAT concentrations. In relation to the DSM, persisting cortical dopamine allows strengthening of all columns associated with both correct and incorrect responses, but only rewarded columns in the circuit are those that will lead to thalamic disinhibition which preferentially strengthens the correct columnar circuit, likely based on STN's excitatory input to the pallidum that leads to inhibition (discussed below) of basal ganglia output associated with incorrect columns. The persisting cortical dopamine may be a major factor in explaining how after repeated object-reward association, the PFC continues to show value-biased responses to objects even in the absence of reward [65]. The rapidly clearing striatal dopamine can allow it to be responsive to short term reward changes (e.g., extinction and reward devaluation).

There is a change in striatal areas associated with overlearning sequences that are viewed as habits. Ceceli and Tricomi [66] discuss structural and functional research showing that caudate and vmPFC are involved in goal-directed actions, while posterior putamen and premotor cortex are involved in habit-like behaviors. Interestingly, a study [67] showed that long-term stress reliably promotes habitual control and was associated with increased putamen volume and atrophy in the caudate and medial OFC. The patterns were reversed with stress is absent. Thus, chronic pain is a long-term stressor and, as will be discussed, some studies show chronic pain to be associated with putamen as opposed to caudate activation. In the next section, the means by which the cerebellum can control habits will be discussed.

In relation to cortical columns, the only meaningful way that the basal ganglia can provide inhibition requires each cortical column to have its connection to specific cells in the dorsal and ventral striatum. The inhibition of columnar circuits will primarily involve frontal action columns with any posterior cortical inhibition occurring after the action columns are involved. In explanation, the feedforward processing from posterior cortical areas involving external and internal stimuli has to be processed as quickly as possible for survival purposes. Logically speaking, inhibition would slow processing speed and be counterproductive. As already stated, non-attended posterior cortical processing theoretically will not impact ongoing responses and there is no need for inhibition of columnar circuits. An example of the importance of uninhibited feedforward processing in the posterior lobes relates to the sensory input to the BLA influences the CeA [68]. From a survival standpoint, it is of value to have immediate defense responses to environmental situations previously associated with negative or dangerous outcomes. The logical conclusion is that emotional responses associated with output from the CeA as a result of posterior cortical (i.e., learned associations related to environmental stimuli) input occurs with no immediate way to prevent them via top-down frontal mechanisms. In other words, when posterior cortical negative emotional memories are activated, there is an uncontrollable (i.e., control requires the subsequent activation of frontal action columns) immediate emotional response that occurs.

An example related to basal ganglia inhibition is saying a particular word aloud requires the word action column to be activated. Prior to activation of its lower order columns, the word column activates its respective cells in the striatum and involvement of the cortex-basal ganglia-thalamus-cortex loop. The thalamic input to the word column and its associated lower-order columns provides columnar activation leading to the word being spoken. Due to learning, which involves strong connections of columns in that circuit, this becomes a high probability neural process. If that high probability response must be inhibited, as in the case the person is to name the red color in which the word is printed instead of saying the word itself (e.g., green), the pallidum maintains its tonic inhibition of the thalamus (likely from STN input), preventing the activation signal from being sent to the cortex.

In relation to posterior cortical columns from a DSM perspective, the basal ganglia inhibition is of importance when an ongoing stimulus becomes irrelevant or needs to be ignored. In that case the frontal action column and/or its associated posterior cortical receptive column can be inhibited via the basal ganglia and allow attention to shift away from the column, thus meeting the same fate (i.e., deactivation without attention) as the multiple other ongoing columnar circuits that are irrelevant to an ongoing response.

Of particular importance to cerebellum activation related to cortical columns is the STN. The STN provides “hyperdirect” excitatory input to the GP and SN that can prevent disinhibition of tonic thalamic inhibition. The STN receives direct input from the frontal cerebral cortex (including ACC) with its output going to the GP, SN, and the pontine nucleus [59],[69]. The input into the GP and SN may functionally provide immediate inhibition of an ongoing response (i.e., columns activating their lower-order columns) if stopping is required or a conflict occurs [70]. Its output to the pontine nucleus allows a connection for the STN to initiate cerebellar involvement. Cacciola et al. [71] provided evidence of strong connectivity of the STN to the ipsilateral cerebellum. The discussion now shifts to the cerebellum.

### 5.3. Cerebellum automatic control

The cerebellum contains about half the total number of neurons in the brain and is involved with multiple cerebral cortical functions, the best known of which is motor functions [72]. Based on the DSM, the cerebellum assumes control of the action columns involved in overlearned behavioral responses, freeing the PFC from using attentional resources for those actions. This is consistent with the conclusion of Balsters, Laird, Fox, and Eickhoff [73] in which the role of the cerebellar cortex is to automate information processes within cortical territories. If accurate, then recurring actions related to pain are expected to become automatic and under cerebellar control. Support for this proposal can be taken from a SPECT study [74] comparing chronic low back pain patients without structural abnormalities to acute back pain patients with herniated discs. Chronic pain patients showed significantly reduced blood flow in the PFC while having increased blood flow in the cerebellum. Additional studies will later be discussed to support this proposal.

Schmahmann and Sherman [75] discussed the cerebellar cognitive affective syndrome in which isolated damage to the cerebellum may lead to both cognitive deficits and emotional dysfunction. It has been shown there is a functional topography in the cerebellum related to motor, cognitive, and affective processing [76]. A resting state functional connectivity study [77], which included self-organizing map approach, provided evidence that functional regions of the cerebellum may be correlated with specific, small cortical regions and is consistent with that expected based on the DSM. Notably, Schmahmann and colleagues (e.g., [78],[79] discussed the proposal that there is a Universal Cerebellar Transform (UCT), such that there is a basic neurological process in cerebellar design regardless of the functions involved. If accurate, there is expected topographical design in the functional circuits among the individual cortical columns, basal ganglia, thalamus, and cerebellum. This view of a common design in each of the functionally connected areas is also consistent with my proposal [36] that the hippocampus has a basic design and purpose in its interactions with circuits of columns; binding parallel cortical columnar circuits for complex memories (e.g., explicit memory) and facilitating the binding of older memories with newer related information. This leads to the conclusion that the variety and complexity of higher cortical functions results from the different information represented by circuits of columns, and not from different modules or different ways of processing information in the cortex and sub-cortical structures.

If there is a UCT, then the best researched area of motor control should serve to explain the manner in which the cerebellum is involved in cognitive and affective processing. In relation to motor functions, both parietal (somatosensory) and frontal cortex project to the pontine nuclei which in turn project to the contralateral cerebellum [59],[72]. As mentioned, the frontal cortex activates the STN which also projects to the pontine nuclei. The cerebellar output in cerebral cortical functions is from the contralateral dentate nucleus to the ipsilateral ventral lateral thalamus, with some evidence that it also projects to the pallidum. The thalamic connections to the cerebral cortex are to the same frontal cortical areas from which the signal originated, forming closed loops. In the same manner that was previously described in which palladial inhibitory input to the thalamus is removed and leads to a cortical column's output, cerebellar input directly or indirectly to the thalamus might lead to activation of a cortical column's output. Recent evidence [61] shows there may be direct cerebellar input from the dentate nucleus to internal globus pallidus (GPi) which could serve as the means by which the cerebellum can disinhibit thalamic to cortex inhibition. Additionally, Cacciola et al. [71], Milardi, Arrigo, et al. [69], and Milardi, Gaeta et al. [80] provided evidence of direct cerebellum connections to the SN. The GPi and SN are the two output sources to the thalamus that provide tonic inhibition to receiving sites. Over time, the related premotor action columns involved with an overlearned response can then be activated directly by the cerebellum to GPi/SN/thalamus to sensorimotor cortex circuit, without any signal originating in the PFC. In that case, the posterior cortical receptive columns' input to the pontine nuclei activate the cerebellar circuits involved in the overlearned response. This explains the previously discussed putamen and premotor activation associated with habits as opposed to the caudate which is involved in the acquisition of learned associations. Habitual behavior is automatic which simply means it occurs independent of PFC columns involved with attention and verbal awareness.

For example, the automatic response (e.g., swinging a baseball bat, dribbling a basketball, etc.) is exact because it theoretically involves the cerebellar modeling of the columnar circuits involved in the original learning of the response. In reality, the automatic response should eventually be better than one made with focused attention due to the strength (i.e., structural synaptic connections among the neurons) of somatosensory and premotor/motor columnar circuits. If one later focuses attention (PFC action columns) to making the automatic response, there may not be the same precision due to a failure to activate the highest-order action column that were previously associated with the automatic response. A possible explanation is that a different, but closely related, action column may form based on slightly different somatosensory columns being activated during the motor response or, if the individual is attempting to alter or improve the response, activating new lower-order action columns. Another possibility is that the verbal decision to change one's automatic behavior involves only the left cortex and fails to initiate the same right cortical columnar circuits that were involved in the original learning of the response. As with a baseball player in a batting slump, the frontal attention to making the response overrides the automatic response controlled by the cerebellum and may result in inferior performance. If the player develops a slightly altered response (e.g., changing the hand position) leading to better results and then rehearses the new response until it is overlearned, then cerebellar control of the new columnar circuit will eventually occur.

The analogy to sports is relevant to pain treatment in that the only way to overcome unhealthy automatic behaviors controlled by the cerebellum is to overlearn a competing healthy behavior. Thus, repeated practice is necessary. For example, simply telling someone to use better body mechanics will have no effect if the person's attention is focused away from doing the proper behavior because it is again under cerebellar automatic control of the less healthy body movements. Just like a baseball player changing his grip on a bat and repeatedly rehearsing the new pattern, the pain patient should spend time repeatedly doing the alternative behavior until it becomes automatic. The same can be true in any actions controlled by the cerebellum, including pain behaviors and other behavioral responses (e.g., teeth clenching leading to facial and headache pain) that may be dysfunctional.

Similarly, all overlearned non-motor cortical functions associated with actions can theoretically become automatic. This can explain why the cerebellum shows increased fMRI activity with so many novel cognitive tasks because it is being activated in case the involved action columns become repetitively used. Thus, from this viewpoint the cerebellum is not involved with initial processing related to new tasks. For example, if one gives the same talk or lecture a large number of times, it can become automatic and not require significant PFC attentional resources. Similarly, overlearned syntax in language can become automatic. In relation to right cortical functions, overlearned analysis of and reaction to others' non-verbal emotional expressions (e.g., facial expressions, voice intonations/volume) can become automatic, as can overlearned visuospatial functions (e.g., traveling the same route between home and work). A recent study [81] provided evidence that, in conflict between goal-directed and habitual control, the cerebellum and premotor areas showed increased activation during habitual control. During goal-directed success, the caudate, dlPFC, and frontal pole increased activation. If automatic control of behaviors is lost due to cerebellar damage, then it requires PFC voluntary control. The task can still be done, but it will be slower and probably with less precision, requiring the need to focus attention to activate the appropriate PFC columnar circuits. The latter point is of significance to changes in cognitive functioning resulting from cerebellar damage, as well as the effects of cerebellar dysfunction in autism and schizophrenia. However, that discussion is beyond the focus of the current paper.

## 6. The DSM and pain processing

Despite the fact that various brain areas are involved in pain processing and there can be morphological changes in some areas (e.g., dlPFC) associated with effective pain treatment, there is an absence of specific theoretical explanations as to how identifying areas informs treatment approaches. Each of the previously discussed brain areas will now be discussed based on the columnar model with the subsequent goal of suggesting how that may inform treatment. As the information is discussed, there will be mention of cortical areas beyond those previously described as associated with any given pain patient. This is based on the fact that experimental designs vary, and the stimuli and subjects (e.g., acute versus chronic pain, new versus well-learned information/tasks) may determine different areas of activation. For example, we [37],[41] made the observation that there are reports of decreased fMRI blood oxygen level dependent (BOLD) response with well-learned tasks and a lack of observable cortical activation (e.g., repetition suppression) does not necessarily mean the columns in a cortical region are no longer involved. Moreover, the automatic control by the cerebellum may negate a column's activation. An additional point is that, based on an individual's idiosyncratic learning history, some cortical areas may show activation not present in other experimental subjects or pain patients.

Based on the DSM, the basis for the involvement of each pain-related cortical region is that a column forms in each region that reflects the summation of information coded by all columns that project to it in AND-gate fashion. In posterior cortical serial circuits, there is a direct progression from lower-order to higher-order receptive columns in serial fashion. Each serial circuit will end with its highest-order column that represents all the information of lower-order columns in that circuit. If there are other posterior cortical serial circuits associated with ongoing processing, the highest-order columns of all circuits project to a common column in other posterior areas (typically referred to as “hubs”). In that case the highest-order column of each parallel circuit is now considered lower-order relative to their common higher-order column. The highest-order columns of parallel circuits are in the medial temporal cortex (i.e., parahippocampal, perirhinal, and entorhinal cortices) which in turn project to the hippocampus, allowing complex memory consolidation (e.g., explicit memory) involving the associated parallel circuits [36]. As discussed, each posterior receptive column has an associated frontal action column. Lateral cortical receptive columns have lateral cortical action columns. Medial temporal lobe receptive columns have medial frontal action columns. Thus, there is a logical pattern of connections predicted based on the DSM.

An example of a “hub” in the frontal area that involves multiple functions is the subgenual cingulate region of the vmPFC. Area 25 has been viewed as a keystone for interoception, emotion, and memory, with Joyce and Barbas [82] describing the cortical connections in rhesus monkeys. The DSM views this as an area of higher-order columns representing the information of each of the inputs. Area 25 connections to the temporal pole, entorhinal, perirhinal, and parahippocampal areas were viewed by Joyce and Barbas as being related to memory-processes. The DSM interprets this in a more specific manner. The area 25 action columns are theorized to be those associated with some of the highest-order receptive columns in the auditory association cortex, temporal pole, and medial temporal lobe structures based on the DSM [36] which project that complex information to the medial temporal lobe prior to hippocampal involvement. Area 25 columns can activate each lower-order action columns connecting to it. The connections to auditory association areas and temporal pole theoretically involve action columns responding to the conspecific vocalizations and sounds involving affective information noted by Joyce and Barbas [82]. The connections with the insula allow action output from Area 25 based incoming interoceptive information which is combined with other information input. Area 25 has also been shown to have robust connections to the amygdala [83]. Ray and Zald [84] suggested the strength of Area 25's connections to the amygdala as its being a particularly important location (of higher-order columns based on the DSM) through which different PFC regions influence the amygdala.

The DSM indicates all intentional and automatic cognitive functions are based on columnar circuits. However, intentional does not necessarily involve having verbal awareness which involves the previously mentioned vlPFC (i.e., verbal interpreter). As will be discussed, intentional volition is viewed as the role of the cingulate cortex. If accurate, it makes it possible for the same mechanisms used by the cerebellum and basal ganglia to control the intent to perform an action controlled by the ACC. In explanation, the signal leading to action may be controlled by ACC (voluntary intentional actions) and cerebellum (automatic actions) via connection to the same thalamic nuclei as the pallidum or to the pallidum itself. The result is to remove or override the pallidum's tonic thalamic inhibition to action cortical columns required for a behavior to occur.

### 6.1. Spinal cord

As previously noted, there is spinal cord nociceptor input to the thalamus and PBN. Therefore, increased nociceptor input is the first potential aspect that may be involved in the transition to chronic pain. There are several recent discussions of the nociceptor and spinal cord mechanisms that can contribute to chronic pain to which the interested reader can refer [85]–[87]. As will be discussed in relation to the DSM, the increased frequency of firing of specific nociceptors and/or the activation of additional nociceptors relayed through the thalamus is proposed to be coded in pIns columns as pain intensity and the motivational aspect is coded in the MCC columns. The PBN activation of the CeA leads to activation of higher-order columns in the mIns that reflect both CeA emotional intensity and pIns sensory pain intensity. As can be seen, it is proposed that the same nociceptor input to the brain is coded cortically as sensory intensity, emotional intensity, and motivational intensity via separate channels. It is predicted that the pIns, MCC, and CeA will be simultaneously activated with the mIns being subsequently activated. These areas appear plausible based on the brain dynamics associated with tactile allodynia [88]–[90], although the cited studies did not provide an identification as to the sequence of activation of the various areas.

Based on the DSM, the primary receiving columns of S1 project to S2 such that S2 columns represent higher-order columns. In relation to columnar theory, it seems possible that the VPI projections to S2 may allow better discrimination of the location and extent of pain signals because it represents a combination of all areas represented by each of the S1 columns and possible relevant input from other areas. This lateral cortical discriminative pathway allows for activation of the parieto-frontal regions of the salience network (attention to the pain), as well as any columns that code for external visual (originating in the occipital lobes) and auditory (originating in the temporal lobes) that may be associated with the pain experience. If a certain somatosensory experience repeatedly results in severe pain (related to S1/S2 columns and the aforementioned nociceptor columns in the Ins and MCC), then similar sensations can lead to the activation of the memory of the situation in which the pain occurred. For example, experiencing repeated extreme pain when engaged in sexual intercourse can contribute to anticipatory anxiety with decreased libido. This may theoretically occur either by thinking about or approaching the stimulus situation resulting in visual columnar circuitry in the occipital lobes leading to activation of somatosensory and/or posterior Ins columns with resultant anxiety (feelings of apprehension) and increased muscle tension. Similarly, one's partner making verbal statements about sex can lead to the temporal lobe activation of the negative emotional memories.

An important point based on the DSM is that the information that reaches the cortex is processed faster in the right hemisphere compared to the left. Therefore, with each of the areas discussed, those in the right hemisphere have fewer columns in the circuit which can explain why the right hemisphere's higher-order columns are preferentially involved in certain functions (e.g., right aIns in emotions). However, if analytical functions and/or verbal processing (e.g., giving pain ratings) are required, the left hemisphere is necessarily involved. This may serve to explain the commonly reported lateral differences in brain activity reported in pain research.

### 6.2. Insula

The Ins has been divided into the anterior (aIns), middle (mIns), and posterior (pIns) areas [91]. Cloutman, Binney, Drakesmith, Parker, and Ralph [92] used *in vivo* probabilistic tractography to map seven anatomically-defined insular subregions. There were anterior and posterior networks revealed. There was strong within-Ins connectivity for both networks, as well as anterior-posterior interconnectivity in the transitional area. The anterior-most Ins areas are connected with OFC and temporopolar regions, as well as to vlPFC. The aIns has been implicated in a range of cognitive processes, including pain perception. The authors noted the pattern was consistent with the functional interpretation that semantic information (temporal pole) and emotional content and valence information (paralimbic and subcortical structures) converge to form a saliency network involved in implementation of goal-directed behavior. The posterior network involved connectivity between the posterior-most Ins areas and predominantly posterior superior and middle temporal areas, including auditory cortex. There was also connectivity with the anterior temporal gyrus and Rolandic operculum. A transitional area involved the dorsal-middle region of the Ins which showed a graduated pattern of anterior-posterior hybrid connectivity between frontal and temporal areas, as well as with Rolandic operculum. The authors noted the connectivity pattern of the posterior and transitional areas may allow for Ins involvement in speech articulation, emotional voice intonations, and singing. The DSM interpretation is that the entire Ins involves receptive columns with the associated action columns being located in the frontal areas. The transition areas to aIns involve hierarchical higher-order column formation, with the highest order receptive columns being located in the most anterior areas.

It is important to note that both positive and negative stimulus information involves the Ins. For example, pleasant touch from hairy skin involves sensory input from unmyelinated tactile C (CT) fibers [93]. Bjornsdotter and Olausson [94] provided evidence of a functional relationship between CT signaling and processing in the pIns. A later study [95] suggested pIns pleasant touch is accomplished by relaying information to the aIns that is connected to emotional parts of the brain. From a DSM perspective, this is interpreted as the columns of the pIns reflecting sensory body location and intensity information that is combined with affective information from other sources (amygdala, hypothalamus) first in the higher-order columns of the mIns prior to reaching the highest-order aIns columns.

The dorsal posterior Ins (dpIns) is proposed to involve interoceptive awareness of bodily states that include graded pain, graded temperature, and dynamic or painful muscle sensation [96]. For example, in pre-surgical patients with intracerebral electrodes who were subjected to thermal laser pain [97], S2 response (evoked potential amplitude) showed gradual intensity changes from sensory threshold to a level next to pain threshold, but a ceiling effect for higher pain intensities. In contrast, pIns cortex did not detect nonpainful laser pulses, but reliably responded to intensity variations at painful levels. Based on the foregoing information, it appears that the primary area of the pIns that receives thalamic input involving somatosensory (VMpo) or auditory input from the primary auditory cortex which involves stimulus characteristics, including magnitude or intensity. This is consistent with the findings of Baliki, Geha, and Apkarian [98] in which they concluded the Ins is a multisensory cortical area for “how much” which includes subjective magnitude assessment of nociceptive information. As the stimulus information moves forward in the Ins it incorporates other input from the amygdala (emotional) and bodily sensations from autonomic activation (e.g., visceral) in columns receiving the subcortical input. The aIns columns represent all the lower-order columnar information which relies on the left vlPFC to verbally label as to type of emotion.

In a study supporting the increasing specificity of Ins receptive information, Zhang et al. [99] provided an assessment of emotion related to auditory processing in the human Ins. The emotion stimuli were voices expressing anger, fear, disgust, happy, sad, and neutral. The structural and functional connectivity data showed two distinct neural networks; the pIns which is primarily connected to visual, auditory, and sensorimotor cortices and the aIns which is primarily connected to both frontal and cingulate cortices and the amygdala. The pIns and Heschel's gyrus (HG) responded most to acoustic features of sound stimuli with HG being shorter in latency, suggesting the pIns may receive input from HG or the thalamus. The amygdala and aIns had stronger responses to emotional features of sound with aIns having a shorter latency compared to the amygdala. A final finding was that progressing from the pIns to the aIns there is an increasing gradient of the emotion selectivity index along with enlarged separability of the emotion types. As was previously suggested, the posterior to anterior information involves progressively higher-order column formation with the most anterior columns representing the categorization of the emotion in a similar manner that the posterior to anterior lateral temporal cortex involves increased complexity (e.g., moving from modality-specific to modality-general) in the visual and auditory streams [100]. In relation to lateralization, the right hemisphere showed significantly stronger connections between the Ins and amygdala and a higher emotion selectivity index as compared to the left. Such preferential emotional processing is explained by the right cortex having fewer columns in its circuits leading to its being involved in non-detailed information that includes non-verbal emotional stimuli analysis and action.

VMpo input to dpIns is arranged somatotopically in the anterior-to-posterior (face to foot) direction [96]. The DSM proposes the somatotopic arrangement involves columns in the same manner as S1, although lacking perception of highly localized body sensations. In explanation, nociceptor terminals end over an area exceeding a dozen millimeters in contrast to mechanoreceptors that are localized across a few millimeters [19]. The C fibers have input to the spinal cord across four to five segments and a single dorsal horn neuron is innervated by nociceptors that cover a large area of the body surface. Therefore, each primary pain column in dpIns is expected to represent a large body area, with columns overlapping (i.e., sharing minicolumns). Where primary pain columns' efferents cross becomes a new higher-order column. As pain stimulus intensity increases peripherally, there can be an increase in nerve cell firing and/or adjacent nociceptor terminals may be activated. Theoretically speaking, with increased firing rate the primary receptive dpIns columns are expected to increase their activation frequency. If additional columns are activated based on adjacent nociceptors, intensity may be reflected in additional higher-order columns being activated. If a higher-order pain column is formed in the close proximity to visceral organs' sensory columns, this can explain the perception of visceral symptoms associated with chronic pain. Activation of an organs' sensation columns send efferents that cross the higher-order pain column's efferents resulting in the formation of a new higher-order column. The new column, when activated, is perceived as a combination of the pain and visceral organ sensation (e.g., gut-retching pain).

The mIns receives input from dpIns and relevant emotional sensory modality input, being connected to the amygdala and hypothalamus [96]. This theoretically allows columns to form which reflect the amygdalar (emotional valance and intensity) and hypothalamic (physiological) information to converge with dpIns information to form higher-order receptive columns that connect to the columns in the anterior insula (aIns). In support of this proposition is a functional connectivity study involving thermal pain [101] in which the aIns has been shown to activate following activation of the dpIns and amygdala. The authors interpreted the findings as simultaneous activation of parallel networks by nociceptive input involving the limbic (PBN to amygdala to aIns) and sensory (thalamus to dpIns to mIns to aIns) networks. The aIns columns theoretically serve as higher-order ones combining the information of both systems, representing the initial aspect of the pain emotional experience tied to a noxious stimulus.

Nomi et al. [102] provided functional connectivity evidence of separate ventral affective processing (involving ventral aIns to orbitofrontal cortex, amygdala, and olfactory cortex) and dorsal cognitive processing (involving dorsal aIns to mPFC and frontal inferior operculum) systems. Both the affective and cognitive aIns systems have common connections with inferior orbitofrontal, occipital, temporal, and parietal cortices. Bastuji et al. [101] note bi-directional connections of the aIns to the various cortical areas, including the dpIns. Similarly, the DSM indicates dIns columns process simultaneous information, ventral columns process sequential information, and intermediate columns involve combined simultaneous and sequential processing. This leads to the theoretical proposition that highest-order aIns columns represent the lower-order columns reflecting the intensity and emotional aspects (both categorized and physiological aspects) related to pain. The highest-order columns connect to an associated medial frontal action column and functionally to other cortical areas (e.g., vlPFC) where that information is combined with other aspects (i.e., the highest-order Ins columns reflecting the combined information of intensity (quantitative) and emotion (qualitative) are now lower-order to the action columns that are formed when the efferents cross with other columns' efferents. This is consistent with Craig's [96] belief that the role of the insula is to provide homeostatic emotional integration, with the aIns (mainly right lateralized) providing subjective awareness of all (including pain) interoceptive emotions and feelings/sensations. The right lateralization appears consistent with the previously discussed findings of Zhang et al. [99] in which the right hemisphere showed significantly stronger connections between the Ins and amygdala and a higher emotion selectivity index as compared to the left. Additionally, in a meta-analytic study [103] of morphological alterations, the right aIns emerged as the region with the highest degree value based on connectivity with other brain areas associated with chronic pain. Craig [96] goes on to note that the aIns and ACC are simultaneously activated by lamina I input (via the thalamus) and provide both “a feeling and a motivation” related to emotions. In their data, Zhang et al. [99] noted functional connectivity between the aIns and cingulate cortices. It is to the motivation aspect the discussion will now focus.

### 6.3. Cingulate cortex

The cingulate cortex can be divided into the posterior (PCC), middle (MCC), and anterior (ACC) divisions. MCC receives nociceptive input from the ILN. Posterior MCC (pMCC) and ACC receive fewer ILN inputs than the anterior MCC (aMCC). Vogt [104] cited research suggesting pMCC and dorsal PCC are involved in orienting the body to all sensory stimuli, including pain. Similar to caudal direction of higher-order columns of S1 to S2 information in somatosensory pain discrimination/localization based on the proximal-distal dimension, the DSM predicts the caudal direction of aMCC receptive information to pMCC involves the formation of higher-order columns involving the general body location of the activated lamina I nociceptors. If accurate, then the ACC action columns form based on the receptive PCC and RSC columns such that the greater the distance from aMCC and pMCC there is increasing complexity of information coded by the higher-order receptive and action columns.

Support for the proximal-distal dimension arrangement of the receptive-action dimension can be taken from several studies. In a connectivity-based parcellation study by Beckmann et al. [105] showed lateral parietal cortex high probability connectivity across MCC and PCC regions with reasonably high probability of connection to posterior PCC (pPCC). Premotor cortex and precentral gyrus had the highest probability of connection with dACC. Vogt [106] noted a key feature of MCC is its role in skeletomotor functions in contrast to ACC where emotion and autonomic regulation are predominant. He also indicated acute nociceptive stimuli leads to activity located mainly in MCC based on fMRI. In a review on the role of the PCC, Leech and Sharp [107] note reciprocal connections of ventral PCC (vPCC) and RSC to medial temporal lobe (i.e., perirhinal cortex, PHC, and entorhinal cortex) which I suggested [36] is the location of the highest-order receptive columns. There are also strong connections from vPCC and RSC (DSM receptive columns) to vmPFC (DSM action columns), as well as reciprocal connection of PCC with both dlPFC and the frontal poles [107]. It was proposed interactions of PCC subregions with other intrinsic connectivity networks to regulate the balance of attention along an internal/external dimension and a broad/narrow dimension (i.e., breadth of attentional focus). It has also been shown the functional organization defining the medial-lateral axis of the ventral temporal cortex is identifiable in the medial parietal cortex during memory retrieval of people versus places. Support for the higher-order nature of RSC was provided by Fischer et al. [108] whose data indicated that landmark representations in the RSC are the result of local integration of visual, motor, and spatial information.

The highest-order action columns of the cingulate cortex are theoretically located the rostral ACC (rACC). Tang et al. [109] noted the rACC is involved in the transition from valuation, then to choice, and then to action. They suggested two possibilities in rACC processing. The first is that information processing may change sequentially across subregions within the rACC from valuation around subgenual ACC, to cognition at the center of the rACC, to action in regions close to the dACC. The other explanation, which they supported, is that different functional processing may be integrated into a central location, or hub, across the entire rACC. Based on primate and human data, they found the connections support their integration proposition. The most rostral intermediate transition zone of the 6 rACC zones analyzed showed the highest number of regions and areas within regions that contributed to the strongest inputs, consistent with the idea it is a hub in the rACC. The other rACC sites had fewer, but multiple and specific, inputs. As discussed, the DSM proposes there is actually a gradual transition from lower- to higher-order columns, but there are inputs from other brain areas as this transition progresses. The highest-order columns interconnecting with the most brain areas is in the rACC.

In a tripartite functional organization of the medial frontal lobe, de la Vega et al. [110] indicated the middle zone involved the cingulate and paracingulate gyri. The four middle subregions were associated with pain and cognitive control. Both dorsal MCC (dMCC) subregions were more strongly associated with working memory and coactivation of other cognitive control regions. The ventral MCC (vMCC) subregions were strongly associated with affect and coactivation of subcortical regions, such as the amygdala and striatum. They noted that vMCC was also associated with reward, in addition to negative affect and pain. They made the observation that aMCC clusters were more strongly associated with decision-making (DSM action columns) than posterior clusters (DSM receptive columns).

A recent study [111] recorded individual neurons in the ACC and pPCC of 2 rhesus monkeys performing a saccadic reward task. They found task-relevant, spatially selective feedback signals in both brain regions. There was stronger selectivity for spatial choice and reward-target signals in pPCC and stronger selectivity for feedback in ACC. However, there was not activity indicating a driving of adaptational patterns or predicting the extent to which feedback and contextual information were used to inform choices. The authors believed the results indicate certain cingulate neurons encode diverse evaluative signals needed for adaptive decision-making, but those signals are integrated elsewhere in the brain to guide actions. Obviously, the DSM indicates the coding is at the columnar level. Shenhav, Cohen, and Botvinick [112] proposed dACC plays a central role in decisions about the allocation of cognitive control based on a cost/benefit analyses to identify the highest expected value of control. A similar conclusion has been proposed by Kolling, Behrens, Wittman, and Rushworth [113] based on the research showing ACC to be linked to commitment to a course of action and exploring or searching for alternative courses of action. They note the multiple signals in ACC reflect updating of beliefs and internal models of the environment and encoding choice aspects, including the average value of choices afforded by the environment.

As can be seen, in addition to its role in pain the cingulate cortex is implicated in attention, memory retrieval, spatial processing, sensorimotor functions, valuation, and decision-making. Based on the information discussed, the ACC appears to determine if, when, and what action should be initiated. Thus, the volition to initiate a specific action at a given point in time is its suggested primary role. Thus, extensive damage to the ACC leads to akinetic patterns in which the patient fails to initiate behaviors spontaneously [114]. If motor actions are required, the dorsal aMCC and pMCC columns connect to the parietal somatosensory columns which activate posterior ACC (pACC) columns that involve the volition to act. The pACC columns in turn activate the pre-SMA and SMA columns associated with the actual motor response. If a specific behavior is to be taken based on reward value, vACC higher-order columns provide the signal to initiate the action columns associated with the desired behavior based on the receptive input from pMCC or PCC columns which interconnect with the receptive columns from the other cortical areas connecting to the action columns of the desired behavior. In each case there is input to the MCC and PCC from the specific receptive columns that, in turn, lead to the formation of action columns associated with the desired behavior. However, the ACC action columns formed by the MCC and PCC columns provide the signal allowing the desired behavior columns to proceed. The ACC may provide activation of action cortical columns via direct activation of the thalamus, or indirectly via its connections to the GP [80] to remove the tonic inhibition of the thalamic nuclei associated with the desired response. Thus, as with the cerebellum (UCT) and hippocampus (binding parallel circuits), the cingulate cortex is proposed to have a singular function. Similar to the basal ganglia dysfunction, inefficient signaling from the ACC to allow specific responses slows the speed and accuracy of ongoing volitional behavior. Support for this aspect was provided by Brockett, Tennyson, deBettencourt, Gaye, and Roesch [115] who showed the ACC is necessary for adjusting action plans at the levels of behavior and single neuron firing.

The DSM explains the pMCC and PCC columns are involved in the receptive information from various inputs and the dACC action columns code for the choice of the cognitive aspects with vACC columns coding for the emotional aspects. Different stimuli (chronic back pain, thermal pain to an extremity) in the cingulate cortex and Ins theoretically involve various cortical columnar circuits that that are located within those regions. Another posterior area, the precuneus, is expected to also show functional differences based on locations, as are all other identified cortical pain regions. Interestingly, one study showed the posterior cingulate and ventral precuneus were the most connected functional hub in the brain [116].

### 6.4. Precuneus

In a resting-state fMRI study, Margulies et al. [117] found three distinct patterns of functional connectivity related to the precuneus. The anterior precuneus is connected to the superior parietal cortex, paracentral lobule, and motor cortex which supports its involvement in sensorimotor functioning. The central precuneus is connected to the dlPFC, dmPFC, and multimodal lateral inferior parietal cortex suggestive of its role in cognitive/associative tasks. The posterior precuneus shows functional connectivity with the adjacent visual cortex. These areas were differentiated from the ventral network associated with the PCC with its connections to the medial temporal cortex, dmPFC, lateral mPFC, posterior lateral inferior parietal cortex, and the lateral temporal cortex. The conclusion was that the precuneus should be regarded as anatomically and functionally heterogeneous.

Based on a literature review of precuneus activation patterns in response to specific tasks, Cavanna and Trimble [118] suggested the anterior region was associated with self-centered mental imagery strategies and the posterior region with episodic memory retrieval. Utevsky, Smith, and Huttel [119] did fMRIs of humans performing three cognitive tasks and resting states. They found systematic state-dependent functional connectivity for only one brain region, the precuneus. During tasks, the precuneus had increased connectivity with the left frontoparietal network and during rest increased connectivity with the DMN. They interpreted this as showing the precuneus has a core role in both the DMN and a variety of other processing states.

The DSM views the precuneus as coding for receptive information with it allowing a convergence of internal and external information. As such, during memory retrieval of naturalistic events it has been shown to use both reconstructive and chronological mechanisms [120]. However, the process allowing a shifting between internal and external processing theoretically requires frontal action columns. In relation to pain processing, dmPFC and dlPFC connections can serve to allow the actual shifting between internal and external processing similar to that previously discussed with the PCC. An important point is that the columns between the identified areas of the precuneus [117] are theoretically those representing a blending of the information of each area (i.e., higher-order), with the associated frontal lobe higher-order action columns connected to the higher-order receptive precuneus columns, This allows the toggling between internal and external states, as well as between sensorimotor, cognitive, visual, and emotional processing. Thus, mental imagery that will later be discussed in the treatment of negative emotional memories can involve a combination of two or more of circuits involving the precuneus.

In relation to pain, the precuneus appears to be primarily involved as being part of the DMN when focused away from pain [25],[26]. In relation to empathy for pain, it has been shown [121] that the precuneus, in addition to the vmPFC, superior temporal cortex, and TPJ, have increased activation in studies involving abstract visual cues indicating whether oneself versus another individual is experiencing pain. As opposed to being associated with empathy, the authors suggest a better explanation of precuneus involvement with this experimental paradigm is the act of mentalizing about oneself versus others which may serve as a prerequisite to empathy.

### 6.5. Prefrontal cortices

The dlPFC, vlPFC, vmPFC, and OFC have each been implicated in pain processing and control. Ray and Zald [84] note the PFC is often divided into 6 broad regions: dlPFC, vlPFC, frontopolar cortex (FPC), OFC, vmPFC, and dmPFC. In relation to lateral PFC, an anterior (rostral)-posterior (caudal) axis was proposed by Badre and D'Esposito [122] in which posterior areas represent stimulus properties and anterior areas are specialized for more abstract operations. A dorsal-ventral axis has also been discussed [123] in which dorsal aspects are specialized for spatial information and monitoring and a ventral PFC for object information used in working memory and decision-making. Recent studies [57],[124] provided information supporting the existence of both of the axes. Within the PFC, sub-regions are highly interconnected via short U-shape fibers, supporting the view that control processes are graded along both axes. To the current author's knowledge, similar axes have not been considered in relation to the mPFC, but some of the studies that will be discussed appear to support comparable axes.

Based on the DSM, the lateral PFC is involved with action involving external stimuli while the medial PFC is involved in action related to internal processing. Based on the proposal that there is a decoding operation of actions columns, then the more rostral areas of both lateral and medial PFC are higher-order to the caudal areas which explains the anterior-posterior axis. Moreover, the dorsal cortex processes simultaneous information (which includes spatial processing and spatial attention in the lateral cortex) and the ventral cortex uses sequential processing (which includes object information and decision making in the lateral cortex) which can explain the dorsal-ventral axis. The intermediary dlPFC involves a combination of sequential and simultaneous processing. If it is accurate that action columnar circuits are the basis of explaining the functions of each PFC region, and then the functional and structural connectivity should be consistent with that proposal. It is further predicted that there should be graded transitions in connections and functional activity within the 6 broad regions of the PFC as opposed to sharp demarcations that would be expected if there were modular patterns of functioning.

### 6.6. Lateral PFC

The dlPFC is involved in cognitive, affective, and sensory processing, with the left dlPFC often specifically mentioned in relation chronic pain [15]. However, the discussions related to pain typically involve general explanations of the role of the dlPFC. For example, Seminowicz and Moayedi [15] note its role in pain is ambiguous in that it has been involved with pain suppression, pain detection, and pain sensitization. In relation to pain and affective processing, it has been suggested the dlPFC has its impact primarily via connections to the medial prefrontal cortex [84],[125] which are discussed below.

In a study on structural connectivity of higher order association cortices, Jung et al. [57] provided information on the dlPFC connections with the parietal and temporal lobes. Notably, intra-areal connectivity in the frontal, temporal, and parietal lobes was high and generally graded in nature, with few to no sharp divisions. This observation is consistent with that expected with a columnar circuitry based on the lower-order and higher-order columnar arrangement proposed in the DSM. In contrast, Jung et al. noted that inter-lobe connectivity was relatively discrete and regionally specific. The dlPFC had inter-lobe connections with superior parietal cortex, intraparietal sulcus, supramarginal gyrus, anterior angular gyrus, and posterior middle temporal gyrus. It was noted this corresponds to the multiple demand system (DSM multiple columnar circuits) proposed by Duncan [126]. Based on the DSM, the dlPFC houses the action columns involved in the salience network as related to pain, and it interacts with the DMN via connections to the mPFC, PCC, and precuneus.

The DSM viewpoint of global (right cortex) and analytical (left cortex) is an important distinction in relation to the dlPFC's role in pain. The left cortex involves language processing is necessarily involved in any tasks involving verbal instructions, verbal expressions (e.g., giving pain level ratings), and verbally-based control strategies (e.g., schemas) related to pain. The right cortex is expected to be involved in non-verbal emotional and pain processing, and experiential and visualization control strategies. An additional point is that whichever cortical areas are required or best suited to make a response, those circuits are the ones activated. This requires that one hemisphere's frontal lobe must have the ability to temporarily inhibit the other side to prevent competing responses [37],[54]. Prolonged interhemispheric inhibition can serve as one possible explanation of the volumetric decreases of dlPFC in chronic pain cases.

In the situation in which there is constant pain, the right cortex is expected to be most activated, particularly when negative emotional states occur. As mentioned, Craig [96] believes the right aIns is primarily involved in emotions and pain and the right aIns functionally (as opposed to structurally) interconnects with the right dlPFC. This theoretically indicates there is right to left dlPFC inhibition. When engaged in verbally-based activities involving the left-dlPFC, there is expected left to right dlPFC inhibition. However, the frequent right dlPFC activation is not necessarily expected to show ongoing increased fMRI BOLD based on what is observed in repetition suppression and overlearning. There is an observed lessening of BOLD response with improved learning of repeated stimulus-response tasks. For example, Hempel et al. [127] found right inferior frontal gyrus and right intraparietal sulcus activation on a spatial working memory task was increased during improved task performance through week 2, but decreased at week 4 when well learned.

The DSM explanation of decreased metabolic activity in overlearned behaviors is that as the cortical columnar circuits solidify connections there are fewer total cells activated within each of its column. This is based on the proposition that the column's information representation involves only the outermost minicolumns of the column [39]. An additional mechanism is that the cerebellum assumes control of frontal action columns of a repeated response which would lead to lessened activity.

A study of acute pain sensitivity in healthy control subjects is relevant to the dlPFC inhibitory discussion. Sevel, Letzen, Staud, and Robinson [128] used dynamic causal modeling to show that thermal pain increases right dlPFC inhibition of the left dlPFC. There is also weakened inhibition from the left dlPFC to the right dlPFC, although there is still left to right inhibition. Notably, subjects with greater right to left dlPFC inhibition showed relatively higher pain tolerance. Although increased right dlPFC activation may be adaptive in allowing acute pain tolerance, with chronic pain the ongoing inhibitory effects on the left dlPFC could be a factor leading to gray matter volume loss based on decreased left dlPFC activity. The important role of both right and left dlPFC columns will be discussed in the treatment section.

### 6.7. Medial PFC

In a large-scale meta-analysis of human medial frontal cortex, de la Vega et al. [110] noted a tripartite functional organization. The posterior zone spanned regions associated with motor functioning, such as SMA, pre-SMA, and motor cingulate. The posterior zone was coactivated with motor regions (primary motor cortex and associated thalamus). SMA showed association with pain processing based on coactivation of S2 and thalamus which the authors suggest involves movements in response to pain. Pre-SMA showed more coactivation with dlPFC and aIns, with a stronger association to cognitive control. The authors noted these findings are consistent with studies suggesting pre-SMA is responsible for more complex motor actions that presumably require cognitive control. In relation to the DSM, the dACC initiates the intent to move signaling to the pre-SMA higher-order columns involved in planning the controlled complex motor actions. This, in turn, leads to the SMA simple movement higher-order columns which activate the lower-order M1 columns involved with the actual movement responses. Some support for this rostral to caudal pattern comes from a study [129] in which a superordinate pre-SMA-basal ganglia loop established task-set selection which, in turn, imposed a unidirectional constraint on a subordinate SMA-basal ganglia loop associated with response selection.

The middle zone discussed by de la Vega et al. [110] involves the cingulate and paracingulate gyri consistent which was previously discussed. The anterior zone was accompanied by coactivation with the DMN, being involved in affect, decision-making, social cognition, and episodic memory. Consistent with the columnar circuitry proposal, their results suggest the anterior mPFC is not a unitary area; instead it is fractionated into functionally distinct subregions. The dmPFC was most strongly associated with social processes and is coactivated with the TPJ, which was previously discussed as being involved with mentalizing. Pregenual ACC showed a less specific functional pattern with moderate associations with affective processes and decision-making and the authors noted it could be a hub region (i.e., DSM proposed higher-order columns involving multiple circuits) of the mPFC. The vmPFC was primarily associated with both positive and negative affective processes with strong coactivation of subcortical areas.

A more recent study by Jackson, Bajada, Ralph, and Cloutman [100] found graded changes in connectivity across the vmPFC with distinct subregions. They noted that both hard and graded connectivity changes appear to exist which is consistent with the columnar circuitry proposal. They found the vmPFC has a graded transition between two areas of distinct connectivity. Both functional and structural connectivity varied along a dorsomedial to ventromedial axis. The dorsomedial end of the axis involved greater connectivity throughout the DMN which included cingulate cortex, precuneus, angular gyrus, lateral temporal lobe, inferior frontal gyrus, and medial temporal lobe. The ventromedial end showed greater functional and structural connectivity involving the ventral visual stream and interconnected multimodal regions which included the anterior temporal lobe, medial temporal lobe, and orbitofrontal cortices. Thus, there was a graded transition in connectivity from a DMN-connected medial wall area to a mOFC region associated with the ventral visual stream. Notably, there was an area of intermediate connectivity between the two distinct regions which the DSM proposes are the higher-order cortical columns representing combined information of the two regions. Based on the DSM, each of the axes involve connections of external (lateral) and internal (medial) cortical processing with the more dorsal aspects involving simultaneous information, the ventral aspects involving sequential information, and the intermediate aspects coding for a combination of sequential and simultaneous information.

### 6.8. Orbitofrontal PFC

As related to pain, the OFC may be most involved with the loss of reward (pleasure) contributing to the symptoms of depression. As with other regions discussed, the OFC can also be divided into functional hierarchal clusters based on resting-states fMRI [130]. The medial cluster has been shown to represent expected reward and decision values, being mainly connected with the mPFC and the PCC. The posterior-central cluster showed negative connectivity to a region of the midbrain, possibly the SN. This was viewed as suggesting a possible role in reward learning (i.e., reward predictor error). The central cluster had connections to the ventrolateral and dorsal striatum and dmPFC. The authors suggest involvement in stimuli (ventrolateral) and action (dorsal) value along with the dmPFC's role in representing and updating action values. Consistent with this, Costa and Averbeck [131] found OFC encoding of variables related to learning and managing explore versus exploit tradeoffs (i.e., choosing between options with known values versus exploring unfamiliar options). Kahnt et al. [130] found the three lateral clusters were connected to the temporal and parietal cortices and dlPFC. It was suggested these clusters involve highly processed visual information tied to regulating value representations. A more recent study Samara et al. [132] evaluated OFC and anterior mPFC using individual data as opposed to group averaging prior to parcellation. The 19 clusters identified grouped together into two larger networks. One involved most medial clusters which have been associated with affective and self-centered information. The other involved OFC and vlPFC which is primarily involved in integration of multi-modal sensory information and coding the stimuli's affective value. The DSM indicates the medial clusters are expected to code for internal processes, while OFC and vlPFC are in a transition area involving a combination of sequential internal (affective value) and external (sensory) processing.

Jackson et al. [100] noted that, despite the debate (largely based on the role of fibers of passage effects in ablation studies) on the precise function of the OFC, it appears to combine unimodal sensory and affective inputs in addition to multimodal semantic information which is consistent with higher-order column formation. Based on other studies they cited, the authors discussed the possible role of the OFC in subjective economic value, such as involving emotions, reward, and autonomic processes related to the generation of affective meaning. In this case DSM theorized higher-order columns involve sequential (selecting relative value) and simultaneous (all possible values) of a context-specific object associated with positive or negative emotional and visceral input that may or may not involve a social situation. The authors also discussed the relatively dorsal medial regions as involved in social cognition. This involves the addition of self-versus-others which requires DMN-related activity. Thus, there may be a consideration of the object's value (whether another person or an inanimate object) in the context of one or more people. The combination of the DMN-related (self-versus-others) and OFC (object's value) involves higher-order columns located between the areas. The higher-order columns involved in social decision-making are expected to be rostrally located (e.g., frontopolar PFC).

A circuit model that includes each of the prefrontal areas related to pain was proposed by Phillips, Ladouceur, and Drevets [133] in an attempt to explain the neural basis for multiple types of emotions. They considered automatic emotion regulation to involve subgenual (Area 25) and rACC, while voluntary emotion regulation involved the dlPFC and vlPFC. The authors proposed the phylogenetically older OFC, dmPFC, and ACC provide feedforward internal state information to the phylogenetically newer dlPFC and vlPFC. The dmPFC was viewed as the conduit through which the OFC feeds value information forward to neocortical regions for decision processes. Ray and Zald [84] noted a flaw in the proposed model in that feedforward and feedback directionality does not necessarily fit with that expected based on laminar connections. The DSM provides an additional concern in that the model only discusses circuits connecting general areas, as opposed to the possibility of multiple circuits within those areas. Based on the DSM, there can be multiple higher- and lower-order columns within the same areas involving bi-directional information flow among areas. However, the Phillips et al. [133] model is a step forward in that it puts forth the concept that the various areas associated with emotions dynamically interact in the perception and regulation of emotions, including those related to pain.

### 6.9. Amygdala

In a study that highlights the potential role of negative affect in the development of chronic pain, Vachon-Presseau et al. [134] tracked brain properties in subacute back pain patients and controls over three years. Of the three identified modules involving the frontal lobes, the only structural connections predisposing to the development of chronic pain involved the dmPFC-amygdala-NAc network based both on white matter and functional connectivity. The white matter connectivity remained constant over the three years as did the functional connectivity at year one. However, the functional connectivity dissipated by the third year. Another finding was that the group developing chronic pain had smaller amygdalae and hippocampi total volumes across the three years, while there was no difference in thalami or NAc volumes. Interestingly, the right amygdala showed greater thinning than did the left. The authors noted that smaller amygdala and hippocampus volumes have been shown in multiple chronic pain conditions [135] which they believed supports the interpretation that pre-existing circuit abnormalities induce risk for chronic pain. The authors noted the BLA portion (discussed below) of the amygdala best matches that involved with thinning in the dmPFC-amygdala-NAc module.

As previously discussed, Cai et al. [21] found that activation of the PBN relays the peripheral pain signals to the CeA and is sufficient to cause negative emotion behaviors reflective of depression, anxiety, and aversion. In contrast, activation of the excitatory pathway from BLA opposes the negative emotion behaviors and induces behaviors of reward. Based on the Cai et al. conclusion that the BLA to CeA circuit may be a top-down mechanism for cognitive control of negative emotions related to pain, the Vachon-Presseau et al. [134] finding of the involvement of the probable smaller BLA volume in the dmPFC-amygdala-NAc network may reflect reduced BLA influence on CeA output related to PBN to CeA activation. An additional point is that the increased static white matter connectivity may reflect a compensatory mechanism to allow increased input and maintenance of the BLA control of the CeA activation. It has been noted that smaller amygdala and hippocampal volumes are commonly associated with negative affective states [136] which, in turn, could be a factor leading to the previously discussed finding that pre-existing depression is a risk factor for chronic pain. Some research [137] has also shown adverse childhood experiences to be related to reduced volume of the right BLA and CeA segments which may also be a mechanism by which early trauma may influence susceptibility to developing chronic pain. Although not a pain study, Johnson et al. [138] showed that unpredictable early life stress led to anxiety-like behavior in mice. Resting state fMRI showing hyper-connectivity between the amygdala and PFC, as well as the amygdala and hippocampus. Hashmi et al. [139] compared fMRI activity in chronic low back pain patients (pain present for over 10 years) and early acute/subacute pain patients with no history of lower back pain on a task involving pain ratings over a 10-minute span. Notably, there were no group differences in pain or depression (none were clinically depressed based on the Beck Depression Inventory) ratings. The early back pain group showed changes in the areas often associated with acute pain which included the bilateral Ins, caudate, putamen, thalamus, and ACC. In sharp contrast the chronic pain patients showed changes in mPFC, OFC, and bilateral amygdala. The early back pain patients were followed for a year with 4 separate fMRI assessments and divided into recovering versus persisting pain groups. When comparing spontaneous fluctuations in fMRI in the persistent back pain group, the changes in assessment 1 compared to assessment 4 showed activity shifting to only the mPFC, putamen, bilateral amygdala, left inferior temporal gyrus, and precuneus in session 4. Notably, the early pain groups did not differ on depression and anxiety measures. Thus, this study supports the role of mPFC and amygdala involvement in chronic pain, but shows the changes are not necessarily related to negative affective states.

Based on the foregoing information, there appears to be a growing literature supporting the mPFC and amygdala involvement in chronic pain. However, the Hashmi et al. [139] study brings into question how negative affective states are related to the mPFC and amygdala involvement. If these areas are involved in most or all chronic pain cases, then a diathesis-stress model is reasonable to consider related to those who have comorbid depression and anxiety. However, epigenetics sheds a new light on the dichotomy of diathesis versus stress in that both hereditary and post-natal factors (e.g., learning history, adverse childhood experiences) may serve as the diathesis and play a role in the possible amygdala morphology. The DSM columnar circuitry of the mPFC likely influence resilience to developing negative affective states and more effective coping with pain. Thus, a smaller amygdala and/or hyperconnectivity of the amygdala and mPFC could be the result of prenatal factors and postnatal environmental factors in the presence of the new pain stressor leading to pain chronification, as well as the individualized development of negative affective states. In that case, the development of a pain condition in some patients may interact with the poor resilience mPFC columnar circuitry connected in association with disrupted circuitry of the amygdalae from both prenatal and postnatal factors, resulting in the worsening or development of undesirable psychological conditions. Based on my experience in providing psychological treatment for chronic pain patients, we mental health professionals see mainly those who fail to adequately cope with their pain condition and have comorbid anxiety and depression. The complexity of factors that may be treatment targets will become obvious when the DSM informed clinical biopsychological model (CBM) is discussed. However, discussion of the complexity of the amygdala is necessary to avoid overly simplistic view of the its involvement in chronic pain.

The amygdala subregions and their connectivity to various brain areas show it to play an important role in all emotions [140], as well as chronic pain. It has been proposed that the amygdala's main role involves the evaluation of the behavioral salience and motivational aspects of sensory stimuli that can engage emotional, autonomic, and motor responses via connections to the hypothalamus, thalamus, and cortex [141]. As related to emotion regulation, the amygdala's connections allow rapid, automatic regulation of emotional behavior [68]. Based on the DSM, the columnar circuits to which the amygdala interconnect structurally and functionally are important in relation to psychological treatments because those are the circuits that can influenced, not the amygdala itself.

The three major groups of nuclei of the amygdala are the CeA, BLA, and superficial (SF) subregions [142]. A parallel model of amygdala functioning is gaining support in contrast to the more predominant serial view. The serial model proposes sensory information converges and is integrated in the BLA nuclei and proceeds to the CeA which initiates the various responses [68]. The parallel model views the BLA and CeA nuclei as independent nodes related to emotional learning which work in parallel to determine aspects of incentive processing in both reward and punishment contexts [143].

In support of a parallel view, Kerestes et al. [142] reported findings of their multimodal evaluation of the amygdala's functional connectivity that were largely consistent with prior resting state [144] and meta-analytic connectivity modeling (MACM) [145] studies showing strong differentiation of connectivity of each amygdala subregion with other brain areas. Commonality was found across all approaches in specific co-activations; CeA nuclei were co-activated with right vlPFC, BLA nuclei were co-activated with bilateral dorsal and ventral mPFC, and SF nuclei were co-activated with the right vlPFC and bilateral NAc. A potentially important finding was that when comparing emotion versus non-emotion studies using MACM, there was evidence of convergence among the CeA, BLA, and SF nuclei in the right vlPFC, in addition to bilateral (greater for left) ventral putamen and NAc. In contrast, the non-emotion studies showed relatively greater coactivation of the left vlPFC with the BLA and SF. The DSM posits the left vlPFC is the location of the verbal interpreter circuitry [37],[53],[54] that involves identification, categorization, and expression of verbal information. Based on an expected right vlPFC global, sequential processing analogue to the left analytical, sequential processing of the vlPFC, the right vlPFC convergence found by Ketestes et al. [142] is viewed to support the existence of an “emotion interpreter” involved in the identification, categorization, and expression of emotional information.^1^

A further differentiation related to amygdala output relates to the central extended amygdala and involves the functional connectivity of the CeA and bed nucleus of the stria terminalis (BNST) [146], both of which coordinate the sensory, emotional, and cognitive responses to threat. In relation to common areas, both CeA and BNST show intrinsic connectivity to: PAG; areas of the midbrain that mediate physiological and defense responses (as discussed by Bandler et al. [147]); midline thalamus (nucleus reunions and medial dorsal); caudate; ventral striatum; hippocampus; and mPFC. The CeA had stronger coupling with sensory processing areas that included ventral posterior thalamus, pIns, aIns, fusiform gyrus, and areas of the past-central gyrus. The authors concluded that this supports the CeA as functionally connected to regions which process visual, interoceptive, and somatosensory information more strongly than does the BNST, which is consistent with a strong role in pain processing. There was also stronger connectivity of the CeA to the right supramarginal gyrus, which is involved in visuospatial processing (which may also be involved as activating parieto-frontal pain salience attention). Relative to the CeA, the BNST had stronger coupling with the ventral striatum, paracingulate cortex, and PCC. As such, the authors discussed the BNST's involvement with attentional, motivational, and self-referential networks involved with conscious appraisal of threat and decision-making. In non-human primates, Oler et al. [148] found tracing studies showed BNST received strong input from CeA with a less robust reciprocal pathway. They believed this shows CeA to be a major modulator of BNST function.

In their analysis of connectivity between anterior temporal cortex/amygdala and PFC, Folloni et al. [149] observed prominent and well-preserved bifurcation along the amygdalofugal and uncinate pathways. The uncinate pathway had previously been shown to be bidirectional between prefrontal cortex and medial and lateral basal nuclei of the amygdala. In contrast, the amygdalofugal pathway is predominantly unidirectional efferent projections to subcortical and prefrontal targets. The projections of both pathways joined in the anterior OFC and the rostral mPFC. Based on the DSM, rostral mPFC and anterior OFC house higher-order action columns. The bidirectional connectivity of the uncinate fasciculus may be viewed as a manner in which the higher-order action columns of those areas represent information of multiple columnar circuits that influence the BLA which incorporate the basal ganglia's inhibitory control and dopaminergic learning mechanisms (discussed next). CeA feedforward information via the amygdalofugal path is incorporated in columns of the mPFC and OFC. The BLA input to CeA can influence its output completing a possible feedback loop.

### 6.10. Basal ganglia

The involvement of the basal ganglia in acute pain is pervasive based on the theoretical proposal that it provides inhibitory control for each cortical column. In acute pain, it has been previously noted that there is both caudate and putamen activation. As has been noted, there is evidence that the putamen as opposed to the caudate showed continued activation with pain chronification over the course of a year [139]. In a study with fibromyalgia patients, the putamen showed increased volume [150]. It is suggested that the reported putamen role in chronic pain is best explained by the cerebellum's control over overlearned actions related to pain. The overlearned actions involving the mPFC-amygdala-NAc circuitry are also expected to fall under cerebellar control.

### 6.11. Cerebellum

Based on the assumption that the cerebellum's role is to assume control of overlearned behaviors, its involvement in pain is at the behavioral level (i.e., action columns). In relation to motor responses, there are two specific ways this may occur. The first involves pain behaviors (e.g., grunts and groans, facial grimacing, profanity) and the second involves habitual behavior patterns (e.g., teeth clenching leading to temple headache and/or jaw pain, poor body mechanics) that may cause or exacerbate pain. In relation to cognitive functions tied to pain, overlearned actions of the left cortex may include verbal rumination, worry, and verbal expressions related to pain. The cerebellum can also theoretically control right hemisphere pain-related actions, such as urges to take pain medications, motivation to remain sedentary, and visualized ruminations. The proposal that the cerebellum removes the basal ganglia inhibition to thalamic nuclei means the frontal lobe action columns' voluntary control of overlearned cognitive, emotional, and motor behaviors via ACC input is not required.

As previously discussed, the DSM indicates the initial involvement of the cerebellum in any new situation reflects its potential to assume control of overlearned actions. If accurate, this means that the cerebellar areas activated with a pain stimulus should give an indication of those cortical areas over which the cerebellum can establish such later control. Diano et al. [151] evaluated cerebellar clustering and functional connectivity in response to mechanical pain. They found three separate clusters of cerebellar areas based on connectivity to sensorimotor areas, cognitive areas, and emotional areas of the cerebral cortex. Similar results of both anterior (sensorimotor) and posterior (cognitive and emotional processing) cerebellum have been reported in studies of pain and motor processing [152], as well as pain and unpleasant images [153].

In a study comparing fibromyalgia subjects to pain-free controls, Kim et al. [154] provided information on altered frontal and cerebellar structural covariance networks using spectral partitioning. Pain induction was done by cuff pain algometry in the lower left leg to preferentially stimulate deep tissue nociceptors. Gray matter volume did not differ between groups in the parietal, occipital, and temporal lobes which is consistent with the DSM proposal that receptive columns are not those affected by cerebellar control. Correlation coefficients were significantly higher in fibromyalgia subjects across cerebellar regions of interest compared to that of controls, while the association strength was significantly decreased in frontal lobe regions of interest in fibromyalgia versus control subjects. In relation to small-world properties, healthy controls demonstrated dense network connections in the frontal lobes while the fibromyalgia patients had dense connections in the cerebellum. Additionally, fibromyalgia patients had dense cerebellar connections with medial PFC/OFC, medial temporal lobe, and right inferior parietal lobe. Those connections were merged to make a distinct submodule in which the severity of depression symptoms was associated with gray matter volume, while white matter connectivity was associated with pain hypersensitivity and clinical pain interference. The authors suggested that in fibromyalgia patients, the mPFC and OFC lose connections with neighboring frontal regions while gaining connections with the cerebellum.

If the DSM based explanation of the cerebellum providing automatic action column control is considered, the indication that the cerebellum involvement with mPFC and OFC associated with pain means the cerebellum becomes the primary driver of actions typically associated with the mPFC and OFC. Therefore, cerebellar control can feasibly lead to the alterations in chronic pain patients described by Vachon-Presseau et al. [134] and Hashmi et al. [139] in which the cerebellar to mPFC is what influences the amygdala as opposed to other areas often associated with acute pain such as the bilateral insula, S2, caudate, putamen, thalamus, and ACC.

Recent studies have shown the cerebellum to be involved in executing goal-directed behavior related to reward [155], associative learning tied to reward size [156], and reward prediction [157]. Thus, when considering the OFC, mPFC, and amygdala involvement in chronic pain the cerebellum may automatically control responses to overlearned situations and impact responses to reward situations. For the cerebellum to assume control of frontal columnar circuits' output based on posterior cortical columns to pontine input, it is logical to assume there has to be a means for deep cerebellar nuclei efference to be involved in proposed [36] hippocampal processing that is associated with parallel cortical circuit binding.

### 6.12. Hippocampus

In a tracing study related to spatial processing in mice, Watson et al. [158] determined connections to CA1 and dentate gyrus (DG). There were no direct cerebello-hippocampal connections, although there were indications of first and second order connections topographically arranged in the well-described modular organization of the cerebellum. There were three main inputs to the hippocampus from the cerebellum. One was from the vestibulo-cerebellum, the second was from vermal lobule VI, and the last was from Crus I. Additionally, the authors found synchronous theta range coherence between the hippocampus and discrete regions in the cerebellum that was related to behavioral context. The Crus I hippocampal coherence showed significant increase over learning trials and became dominant after the acquisition of a goal-directed behavior. In their discussion, the authors noted that Crus I is anatomically and functionally associated with PFC. The overall results are consistent with the cerebellum developing control of well-learned behavior associated with parallel columnar circuits and the hippocampus.

In my discussion of the hippocampus as related to the DSM [36], I suggested that dentate granule cell neurogenesis is related to binding of new parallel cortical circuits with the highest order receptive columns being located in the perirhinal and entorhinal cortices, both of which are first order connections to the hippocampus as described by Watson et al. [158]. Thus, there is the means via cerebellar input by which it is directly involved in its connection to the highest-order receptive columns that provide input to the dentate gyrus. That suggests that the cerebellum's circuitry that is connected to the associated thalamic, cerebral cortical, and basal ganglia circuits is being primed from the initial stages of learning to assume eventual automatic control if there are repeated learning trials. That is expected to include each cortical circuit related to chronic pain.

In a study of chronic pain in mice, Apkarian et al. [159] provided evidence that downregulating hippocampal neurogenesis could reversibly diminish (pharmacologically or in transgenic mice) or block (ablation) persistent pain, while upregulating neurogenesis resulted in prolonged persistent pain. The authors concluded that neurogenesis learning mechanisms are involved in the emergence of chronic pain. Of note in the study is that the transgenic mice with decreased neurogenesis exhibited heightened depression but dramatically decreased postinjury pain behaviors.

I posited [36] that the role of mature dentate granule cells is to connect to the highest-order medial temporal cortical column that represents all lower-order columns of parallel circuits. That can explain the necessity for ongoing neurogenesis in that it allows the formation of complex memories throughout one's lifetime. The new granule cell projects to the other hippocampal areas to initiate the reverberating circuits among the thalamus, cortex, and basal ganglia (and cerebellum based on Watson et al. [158]) for strengthening synaptic connections. Therefore, if the only role of the hippocampus is to strengthen circuitry connections, then its role in chronic pain should be at that level. If parallel cortical columnar circuits cannot be bound, then only disconnected serial circuits are involved in pain processing. In that case, the brain response patterns to the presence of chronic pain should remain as that observed in acute pain as opposed to the transition to that associated with chronic pain. Additionally, only simple pain behavioral responses should remain and remove all associative memories tied to the pain. Therefore, the ongoing pain responses are related to thalamic and PBN input. In relation to the thalamically-connected S1, S2, MCC, and pIns columns, only those in direct line to higher-order receptive and action columns may consolidate based on repeated stimulus input or re-entrant processes. It seems likely that with sleep, that direct connections may be disrupted because there would be no hippocampal memory consolidation processes. The ongoing pain-related PBN input to the CeA is expected to lead to the negative emotion behaviors associated with the serial circuits activated by the CeA [21]. The result is that heightened depression-like behaviors occur due to constant pain stimulus input with the absence of learning-related cortical to BLA circuitry to attenuate the CeA output.

In a study of abnormal hippocampal functioning related to chronic pain in both mice and humans, Mutso et al. [160] provided support for the role of learning and memory. Chronic pain mice showed decreased neurogenesis and altered short-term plasticity compared to sham surgery controls. It is of note that there was no direct manipulation of hippocampal neurogenesis as in the Apkarian et al. [159] study, so the decrease occurred solely in response to chronic pain. The authors discussed molecular factors that may influence the reduced neurogenesis. However, the question remains as to whether this is the result of maladaptive (e.g., proinflammatory cytokines) mechanisms suggested by Apkarian et al. or perhaps the result of decreased need for new granule cells (or both). The latter situation could occur because the pain limits new learning resulting from engaging new environmental stimuli or control of attention mechanisms dominated by pain-related columnar circuits. In relation to humans, Mutso et al. [160] found chronic back pain and complex regional pain syndrome patients had significantly lower hippocampal volumes compared to non-pain controls and knee osteoarthritis patients.

In a study comparing subacute back pain patients and chronic back pain (over 10 years) patients compared to non-pain controls, Mutso et al. [161] found weak, but widespread, increases in hippocampal connectivity with the pain patients. The subacute pain patients were followed for a year and divided into those with persisting pain versus those who recovered. The persistent pain patients showed large decreases in hippocampal and mPFC connectivity during the last assessment compared to the increased connectivity during the first assessment. The authors believed the results were consistent with a significant increase in pain-related memory formation and consolidation during early stages of pain, followed by possible ongoing pain-related activity that could compete with non-pain-related memory processing. They felt this was consistent with their prior hypothesis that chronic pain is a state of continual learning coupled with an inability to eliminate aversive associations.

The DSM proposal that the cerebellum assumes control of premotor and putamen activation related to habitual pain actions may result in a reduction in hippocampal activity because it would bypass medial temporal lobe input to the hippocampus. In that regard, I discussed [36] the belief that relevant hippocampal cells activate any time the original well-established cortical circuitry is activated. Based on my current analysis of the information discussed in this paper, this may not always apply based on the assumption that the cerebellum models its circuits to coincide with the lower-order lateral (e.g., motor response) or medial (e.g., an urge) cortical action columns involved in the output from the complex parallel circuits associated with an overlearned response. If accurate, the complex higher-order receptive and action cortical column circuits are no longer required for the overlearned action. The result would be frequent cerebellar-controlled overlearned pain-related actions and fewer new non-pain complex memories being formed. There would be less overall input to the hippocampus with adaptive reduction of new dentate granule cells.

### 6.13. Reward system

Given the focus of the current paper on the cerebral cortical system in chronic pain, it is believed the reader will now have gained a sufficient understanding of the applications of the DSM in explaining various aspects. Therefore, no detailed discussion of the reward system will be presented. The interested reader can refer to recent works on pain as related to the reward system [162],[163], including specifics on the NAc [164], PAG [165], and habenula [166]–[168].

At this point in the discussion, it is hoped that the reader has concluded that the time for modular discussions of the brain as related to pain needs to end, with a shift in focus to cortical circuits. As related to psychological treatments with pain, the prior brain structural and modular discussions have provided no insights as to why specific treatments may have effects, and have certainly not provided any novel recommendations on where the focus of treatment may be most effective. The discussion will now shift to the clinical applications of the DSM, including information on three treatment target areas that are not commonly discussed. Those are effectively dealing with influential interpersonal relationships, addressing influential negative emotional memories, and dealing with loss-related depression.

## 7. Clinical biopsychological model

The right and left hemispheres are viewed as semi-independent functioning units in which interhemispheric connections are to the same cortical areas (e.g., parietal to parietal, frontal to frontal). Although there are columns in the right hemisphere with corresponding columns in the left that represents identical information (cf., Kaschube, Schnabel, Wolf, and Lowel [169]), there should be many more columns in the left that do not have a corresponding right hemisphere column. This makes logical sense if one considers the fact that there are more columns in the left involved in processing information that allow more details. Additionally, the closer in proximity to the primary sensory receiving areas the more the left hemisphere columns have an identical right hemisphere column. This simply means that initial stimulus processing for both hemispheres is largely identical. For those familiar with Lurian [34] theory, this is consistent with his view of the cerebral cortex in which lateralized primary zones are the most similar, the secondary zones are increasingly dissimilar in information being processed, and the tertiary zones are the most dissimilar in information processing.

The right cortex processes information in a global manner which allows it to be relatively faster in processing speed than the left, though the right side has less capacity for fine detail in both analysis and response. Global processing results from relatively fewer columns (compared to the left cortex) in the circuit from the time of sensory input to behavioral response on the right side. Of particular relevance to psychotherapy is the fact that interpersonal interactions rely heavily on non-detailed voice intonations and facial expressions that the right cortex is best suited to handle. This means that the non-detailed emotional and interpersonal sensory and motor memories are stored in the right hemisphere where the processing originally occurred. Thus, many of the sights, sounds, and touches associated with negative emotional memories, including traumatic ones, are stored in the right posterior lobes. The right cortex is proposed to be responsible for our interpersonal “native emotional language” (analogous to the native spoken language of the left hemisphere) leading to our “personality” characteristics. Thus, it is theorized that there is a left vlPFC-located verbal interpreter and a right vlPFC-located emotional interpreter, each of which is comprised of independent circuits of columns. The use of the word “interpreter” is for descriptive purposes as related to the functional role of the areas as opposed to suggesting these are modules. The left cortex involves analytical processing, since it has many more columns involved in its circuits. This means it can handle much more detail in processing, response, and memory storage.

An important point is that a primary goal for all human behavior is to activate positive emotions and deactivate negative ones. This is consistent with the psychodynamic concept of the pleasure principle, as well as positive and negative reward in behavioral formulations. Based on the need for survival, it is important to store information on what situations/factors lead to emotional reactions, which means that the stronger the emotional response, the more enhanced will be the association memory (DSM's selective arousal system). When emotional memories are activated in the cortex, the columns connect to subcortical structures (e.g., amygdala, mesolimbic dopaminergic pathway, hypothalamus) which in turn activate the physiological and motivational aspects of emotions. Because brain structures are paired, then both the right and left cortices have their own respective subcortical structures. This means each hemisphere is expected to have the capability of producing both positive and negative emotional reactions.

The hypothesized cortical influence, notably involving the sensory, or receptive, cortices, on the amygdalae influences proceeds as follows. Cortical input to the BLA leads to output from the CeA and BNST. There are two contributory pathways of note from the CeA [170]. The first results in sympathetic nervous system activation (“fight-or-flight symptoms”) via the lateral hypothalamus/perifornical regions to interomediolateral nuclei in the spinal cord to the sympathetic ganglia. This results in the rapid physiological reactions associated with fear, anger, and anxiety. The other tract activates the slower, but longer, duration effects and proceeds from the periventricular nuclei of the hypothalami to the hypothalamic-pituitary-adrenal axis release of stress hormones. Either the right or left sensory cortices can activate the psychologically-related physical symptoms (e.g., panic attacks, pain disorders, gastrointestinal disorders) impacted by these systems. However, it is believed the right hemisphere is the most influential in negative affective states and personality disorders.

The interconnections of other frontal cortical areas with the verbal interpreter allow the action columns to directly manipulate the associated posterior cortical receptive columns, providing a means to voluntarily control affectively undesirable sensory columns and activate alternative sensory columns (i.e., logical analysis and alternative interpretation or explanation). This same verbal interpreter is also responsible for the expressed words used in labeling emotions. Of particular importance is this region's role in allowing for an internal verbal dialogue. Based on the DSM, actions can be done only by the associated frontal columns. Since verbally thinking to oneself is an action, it must theoretically involve the left vlPFC.

As theorized by Gazzaniga [50], the “interpreter” attempts to make sense of things. He considers it a device, system, or mechanism that seeks explanations for event occurrence. He saw the advantage of an interpreter as allowing more effective coping with similar future occurring events. Notably, Gazzaniga viewed it as only one of the cortical “modules” that exist. Moss [51],[52] went one step further and described it as being involved in all verbal-thinking. This could be as simple as reading words in a text and as complicated as writing a detailed theoretical paper. This is not in conflict with the belief that the sensory processing and memory storage of the words is in the left posterior cortical region; only that when one is actively using the words, it is done in the vlPFC.

The reason the specification of the location of the verbal interpreter is important relates to the fact that only information being processed cortically in functionally connected circuits can be recognized and influenced by this area. Thus, left intrahemispheric processing is much more likely to interconnect to the verbal interpreter than would right interhemispheric processing. In fact, the only expected direct interhemispheric projections to the left vlPFC are from the right vlPFC. If, as has been suggested [53], the non-detailed sensory aspects of emotional memories are located in the posterior regions of the right cortex, there is no means by which verbal-thinking can directly access, label, and influence these memories due to the absence of direct connections. Thus, based on this theoretical model, the left verbal labeling of emotional experiences of the right hemisphere is actually educated guesswork based on experience.

Because both hemispheres are similarly designed in terms of columnar processing patterns, it follows that what is true of one side of the cortex is true of the other side. Since the verbal interpreter is aware of left hemisphere language functions, there is a tendency for therapists and patients alike to see those functions as natural. For example, we verbally learn new concepts and incorporate these with our pre-existing views and concepts tied to many things in our lives, but fail to recognize that the new ways in which one verbally thinks about matters is analogous to proposed emotional “memory reconsolidation.” [171] In other words, the same cortical mechanisms of change occur tied to “memory reconsolidation” in both hemispheres. The vlPFC columnar circuits are responsible for the new ways one verbally thinks. As previously mentioned, it is logical, based on the DSM, to conclude the right vlPFC has an “emotional interpreter” circuitry involved in the categorization and vocal expression of emotional information. Additionally, columnar circuitry in mPFC theoretically determines changes in one's self-perception. It should then be no surprise that the right cortex is fully capable of learning new concepts and incorporating these with those that pre-existed. This involves the action columns in the right frontal cortex. Notably, if the new information involves right cortical columns with emotional associations, the so-called “reconsolidation” is perceived as a change in emotional processing, not a change in verbal processing.

Repeating words in one's head involves activity in the verbal interpreter (i.e., action columns in the left vlPFC). Taking the same concept, it is possible to apply it to a different action, such as watching someone using a hammer and thinking about performing that action. In that case the columns in the motor planning area located anterior to the hand area of the primary motor strip and the area posterior to the hand area of the parietal primary somatosensory strip are activated. Just as I can repeat words in my head to fully comprehend what is said, I can repeat the physical action in my head to fully comprehend the action involved with using a hammer. This is the nature of the parieto-frontal mirror neuron system based on columnar theory.

Based on the DSM, the hemisphere which can best respond to a given situation is the one that assumes control of the response. This means that the controlling frontal lobe must be capable of inhibiting the other frontal lobe to prevent a potential competing, incompatible response. As previously discussed, the basal ganglia are proposed to control columnar circuit inhibition. Although there can be interhemispheric communication among basal ganglia structures controlling the process, there is evidence that in relation to the NAc shell there are both ipsilateral and contralateral projections from the vmPFC [172]. Contralateral projections allow a possible explanation for interhemispheric inhibitory control since it can account for the quickest possible direct inhibitory mechanism limiting the effects of frontal columns in the contralateral cortex. The Bossert et al. [172] study involved context-induced reinstatement of heroin seeking following extinction. They suggested that only a small minority of context-encoding mPFC neurons mediate context-induced reinstatement. The context-encoding mPFC “neural ensemble” is comprised of neurons that project to both ipsilateral and contralateral accumbens' shells. The DSM holds that the “neural ensembles” are cortical columns encoding the contextual information based on interconnections with other cortical circuits. Regardless of the exact connections and mechanisms, interhemispheric inhibitory control leads to several relevant conclusions tied to psychological symptoms in negative emotional states.

The rapid processing in the right hemisphere allows faster response patterns. With situations creating negative emotional responses, the right frontal area is quickly activated, with potential inhibitory influence on the left frontal lobe. In situations resulting in inhibition of the left vlPFC activity, the perceived symptom would be impairment in one's verbal-thinking ability (e.g., finding the words one is trying to say, detailed memory recall). This can result in problems accessing verbally-based information (e.g., test phobics knowing the material but being unable to access it due to anxiety while being tested) and impaired attention for details. It does not matter if the right hemisphere's excitatory receptive sensory processing is the result of a new aversive environmental stimulus or the activation of significant sensory negative emotional memories (or both), there is activation of the right frontal lobe and its inhibitory influence on the left vlPFC occurs. If the right frontal lobe does not effectively address a situation, the slower left cortical posterior excitatory activity has been ongoing and activates the left frontal lobe. The left frontal lobe has the ability to exert inhibitory influence on the right frontal lobe to allow it to assume control of the ongoing response. Such parallel processing allows the two hemispheres to be involved simultaneously in any given situation and to employ the most efficient and effective solution. However, there are many situations which are beyond the control of either hemisphere. In this case, such as an unexpected and uncontrollable emotionally overwhelming event, both frontal lobes receive ongoing excitatory sensory input which maintains increased inhibitory influence on the other frontal lobe. This can explain perceptions of emotional numbing and depersonalization. In this case the inhibition creates attenuation of input from the opposing hemisphere, to some degree being functionally detached. This can also account for the observed electroencephalogram pattern reported in some studies of depressed patients in which the right frontal activity is relatively greater than the left [173], though both frontal lobes often have decreased activity based on theorized reciprocal inhibition.

An additional aspect of the mutual inhibitory influence of each frontal lobe relates to emotional expressions from the right side. When well rested and/or in a calm state, the left frontal cortex can most effectively inhibit right frontal emotional expressions (i.e., logical analyses are dominant). However, with fatigue or if in an emotionally aroused state, left hemisphere detailed processing takes greater effort and there is a growing inhibition from right to left frontal cortex. The result is that right frontal control and responses are more likely. This may involve poorly regulated responses, such as attack (verbal or physical), escape/avoidance, or engaging in addictive behaviors.

An important point of the DSM is that each side of the brain stores its own memories tied to the processing used. This means that non-detailed sensory emotional experience memories are stored in the right posterior lobes, while the non-detailed action or response memories are housed in the right frontal lobe. Due to the fewer number of columns between sensory input and behavioral output, right cortical complete circuit reception-leading-to-action memories are formed faster than in the left. Developmentally speaking, and due to fewer connections in the circuit, the initial right hemisphere's emotional memories are formed prior to the left hemisphere's verbal memories. Depending upon whether there are early positive or negative experiences tied to relationships, these memories will be the ones activated in future situations with others. Just as individuals learn a left hemisphere native verbal language that remains for life, they learn a right hemisphere native emotional language that remains for life. Thus, the sensory emotional memories leading to the activation of positive and negative reactions (i.e., what feels positive and what feels negative to each person) in response to the behavior of others are stored in the posterior right cortex. The behavioral expression (i.e., one's “personality”) involves right PFC columnar circuits. Therefore, one's interpersonal relationship behavior patterns are largely a function of right hemisphere processing and memory. Based on the previous discussion of the cerebellum's control of overlearned actions, it is expected that relationship behaviors become automatic. If accurate, then left and right lateral and medial PFC attention and control are required to engage in behavior patterns inconsistent with one's native emotional language that automatically occurs via cerebellar mechanisms. More details on proposed behavioral patterns will be discussed below in the ongoing factors section.

The CBM indicates that there are three sources leading to negative emotional states either singly or in combination. These are ongoing situations, activation of negative emotional memories, or failure to activate positive emotional memories. However, it is also necessary to look at each of these three sources in relation to the impact on each hemisphere and the congruence of frontal activity between the hemispheres. Interhemispheric congruence simply refers to the degree to which there is consistency of the analysis and response of each frontal lobe. The greater the inconsistency, the greater the perceived internal conflict in relation to the behavioral response generated from each hemisphere and the greater the inhibitory input received from the other frontal lobe.

Based on the theoretical formulation that all receptive/sensory information processing involves a feed-forward excitatory process, the posterior lobes can be considered passive. The posterior lobes cannot control environmental sensory stimulus input and resultant processing. As previously discussed, sensory cortex can directly activate subcortical structures such as the amygdalae. With subcortical activation, both the sympathetic and parasympathetic systems, as well as the mesolimbic dopamine pathway, can be influenced. Thus, passive does not mean that posterior lobe processing lacks broad ranging active influence in the brain and body. Therefore, right posterior cortical processing can result in autonomic physiological changes (e.g., decreased blood flow to the gut, increased heart rate) without the involvement of the right frontal lobe. This leaves the left vlPFC verbal interpreter disconnected from the processing and associated effects of the right posterior cortex. In the presence of significant negative emotional memories in the right posterior cortex, there can be both subtle (e.g., decreased gastrointestinal blood flow) and noticeable (e.g., rapid heartbeat) physical symptoms, often without verbal recognition or awareness of why the symptoms exist. This can account for the manner in which anxiety and other psychophysiological symptoms (including those contributing to pain, such as muscle tension) can emerge in the absence of verbal “conscious” awareness.

In reference to effective psychological treatment, the frontal lobe action columns are necessarily engaged. Whether practicing relaxation or mindfulness procedures, or engaging in non-directive therapy dialogue, the action columns are responsible for producing the behavior. However, even in the same hemisphere the frontal columns do not necessarily interconnect. Recall that the developmentally earliest frontal lobe columns form in association with and are connected to the corresponding intrahemispheric posterior columns leading to the same type of information coding (e.g., medial cortex involves internal, self-referential information while lateral cortex involves externally related information). The DSM is consistent with there being multiple frontal attention circuits in different frontal areas, both medially (DMN) and laterally (dlPFC for spatial attention, vlPFC for speech attention). Whichever columns are required in a given task are the ones that activate. This means that different areas in one frontal lobe are responsible for different actions, often with no connections among those areas allowing for verbal interpreter awareness even when it involves other regions of the left frontal lobe. Additionally, action column circuits code for all verbal and non-verbal behaviors, including the maladaptive behaviors observed in patients. This means that treatment based on the CBM can be evaluated on the basis of which frontal columns are involved with a given approach or technique, and whether a given treatment is addressing maladaptive sensory memory processing (e.g., panic attack tied to an external stimulus) and/or maladaptive action processing (e.g., escaping an innocuous situation associated with the panic attack). Therefore, truly comprehensive treatment involves the inclusion of all relevant bilateral frontal areas.

## 8. Ongoing situations

There are a wide variety of situations that have the ability to create negative emotional states. Except in early infancy, no sensory processing and associated responses can be considered independent of memory since learning and associated memories begin early and continue. However, within the context of psychopathology treatment, it is possible to address a client's response pattern to current or anticipated situations employing strategies that require no attempts to deal with past memories. The most obvious theoretical orientation in this regard involves behavioral (e.g., stimulus control, reinforcement contingencies) and cognitive-behavioral (e.g., self-talk, schemas, mindfulness) treatments.

Notably, it is possible to consider case conceptualization to be an ongoing treatment procedure in that it provides a new schema to explain the development, maintenance, and proposed treatment of a patient's problem. This is considered a critical component of the CBM approach, typically occurring by the second session. At face value it appears that a conceptualization is only influencing the left cortex since a verbal explanation is involved. However, the verbal descriptions may lead to visualization which can directly influence the right cortex. Additionally, as the left hemisphere recognizes logical and reasonable explanations as to why problems are being experienced, the less there will be perceived internal conflict. In this case, there is improved congruence between the hemispheres as a result of the left frontal recognition that perceived problems resulting from right hemisphere activity are logical and sensible, with concurrent reduction in PFC interhemispheric inhibition.

As will be evident in the discussion of each hemisphere and interhemispheric congruence, it is not possible to ever consider either hemisphere as completely independent of the other. The smooth coordination among various intrahemispheric and interhemispheric cortical circuits happens in fractions of seconds which allows the emergence of what appears to be a uniform mind. However, the DSM-based CBM allows a way to dissect the components contributing to the emergent mind in psychotherapy. Verbal-thinking represents only one particular function involving the left vlPFC. Although this appears to be uniquely human and a very powerful function, it should not be considered “consciousness” since this is only one of many ongoing frontal lobe actions. Instead, the term “consciousness” may be better defined as related to the ACC which is the proposed source of intentional volition. The ACC controls activation of the outputs of cortical action columns, based on receptive column information, that allows meaningful external and internal interactions. Consistent with Gazzaniga's (2010) view on “emergence” in defining the “mind,” this definition views all frontal circuits as potentially involved in meaningful interactions. The action columns that can best address an ongoing situation are those that assume control, whether or not there is verbal-thinking influence or awareness.

When confronted with novel, unexpected, or threatening situations, the right hemisphere is capable of the quickest cortically processed response. Obviously spinal reflexes (e.g., withdrawing one's hand from a hot object) and subcortical orientation responses (e.g., looking toward the source of a sound) are the fastest behavioral patterns, but the right cortical processing allows for the rapid generalized decision of choosing to freeze, fight, or flee. Contextual cues are influential since the right cortex efficiently processes them. For example, the process variable [174] of therapist “warmth” is proposed to primarily involve the client's right cortex. Non-detailed therapist behaviors such as voice intonation, body position, facial expressions, and eye contact are keys in conveying warmth and acceptance.

Imagery and experience are the two basic ways of directly influencing the right cortex, though in psychotherapy this is typically prompted by therapist verbal interactions and directions with the patient's left hemisphere. Although guided imagery may immediately come to mind as a treatment approach, the use of metaphor and analogy are also ways to evoke mental pictures that allow communication with the right cortex. Thinking spatially involves the dorsal, simultaneous processing parietal lobes interacting with dlPFC. When spatial visualization occurs, the right parietal lobe must be involved. Both parietal lobes are accessed in therapy when describing an overarching model to allow the patient get “the big picture” or “see the forest” prior to giving each of the components. If the patient later has a situation in which the model is applied, the bilateral frontal action columns are employed. If the patient later explains the model to someone else, there is right hemisphere activation and congruent left hemisphere frontal action column involvement. Herein is a prime example of the importance of the level of overall cortical involvement. If the patient only pictures the model (i.e., right posterior involvement), there is very limited frontal activation expected. As a result, no impact on the patient's subsequent emotions and behavior would be expected. Upon successful application of the model in a situation, there is involvement of the right frontal lobe and the patient will likely feel improved understanding and control as a result. This application has its main impact in the right hemisphere and can lead to long term improvement with continued application. In the event that the patient teaches the model to someone, there is involvement of the action columns in the left hemisphere which can allow even better detailed application by the patient. This allows improved interhemispheric congruence since both sides are in concert. Thus, the patient both applying the model and verbally teaching it results in bilateral frontal lobe involvement and theoretically is expected to increase therapeutic impact.

To assist in understanding this, it is possible to use classroom learning as an analogue. Passive learning (e.g., listening to a lecture) has primary effects with receptive columns, while active learning is a function of the frontal columns (e.g., both studying and then applying or teaching it). The DSM clearly indicates passive learning results in formation of associated frontal columns. However, if only briefly used (e.g., long enough to take a test or simply converse about a new schema with the therapist), the integrity of the new columnar circuits will be lost due to disuse with the material being forgotten. I proposed [35] that early long-term memory is chemically-based (e.g., ion concentration changes, increased neurotransmitter stores) with later long-term memory being structurally based due to the formation of synaptic connections. If there is failure to actively use the involved columns, the more permanent structural connections never form and there is a reduction in increased neurotransmitter stores. The result is that the new memory circuit (i.e., upstream columns activating downstream columns) is lost (i.e., the physiological definition of forgetting). As noted by Theobald et al. [175] in relation to science, technology, engineering, and mathematics courses, active learning is grounded in constructivist theory which proposes that humans learn by actively using new information and experiences to modify their existing models of how the world works.

All psychotherapy necessarily involves the left hemisphere. This is due to the reliance on verbal communication. The verbal behavior of the client is generated by the left verbal interpreter. With adequate case conceptualization which outlines treatment, the patient's verbal interpreter has been provided with an organizational scheme which can be used to understand the therapy process, including expectancies of the patient's own behavior in the therapy situation. Action columns of the left cortex are always involved when a patient logically decides to remain in a situation leading to perceived emotional distress. It is important to emphasize that any time a patient finds a discussion in therapy leads to emotional distress, the voluntary choice to continue the discussion must involve the frontal action columns. Thus, the finding of frontal activation should not be considered to be exclusive to certain cognitive–behavioral approaches. If this is accurate, then all forms of effective psychotherapy (both within session and employing techniques in the real world) involve a form of exposure therapy and similar heightened frontal metabolic activity is expected to occur in the areas controlling the new responses (i.e., new columnar connections). This means the left verbal interpreter may be responsible for the decision to remain in a distress producing situation, but may not show fMRI changes since the verbal-thinking does not involve the learning of new words. Metabolic changes are expected in the right frontal region involved in the immediate experience of remaining in an uncomfortable situation, in addition to any newly involved left frontal regions.

In support of this possibility are the data from an exposure study by Hauner, Mineka, Voss, and Paller [176] involving spider phobics. With successful treatment there was an increase in right dlPFC metabolism. However, at the six-month follow-up, this was no longer evident. The authors concluded that “up-regulation of dlPFC processing, as observed in the short-term, was not essential for maintaining either long-term therapy gains or long-term amygdala/limbic responses to phobogenic images.” (p. 9204) As previously discussed in relation to new versus old learning, a reasonable alternative explanation is that the initial post-treatment rise in right dlPFC activity was essential to allow the new memory consolidation (i.e., new action columns). After the new columns and their interconnections were effectively consolidated, the general activation decreases. Importantly, this interpretation says that the right dlPFC columns are involved in both the acquisition and maintenance of treatment effects.

When there is consistency of bilateral hemispheric information processing and analysis, perceptions of internal conflict lessen regardless of the emotional state. Thus, an individual in an emotional state who verbally thinks and emotionally feels that it is reasonable and acceptable to have the experienced emotion has a high degree of interhemispheric congruence. In relation to a process variable described by Rogers [174] that is conveyed by the therapist, both the patient's right and the left posterior lobes are impacted in “genuineness.” In this case, the therapist communicates truthful and consistent verbal (left frontal) and emotional (right frontal) messages to the patient, based on verbal content and with the “heartfelt” aspect of the accuracy being conveyed by the therapist's non-verbal behaviors. The result for the patient is to process the consistent receptive information in both the right and left posterior cortical columns followed by the associated frontal columns being activated. Because the frontal columns of both hemispheres have consistency, the patient will experience minimal conflict. In relation to the process variable of “empathy,” the accurate verbal labeling of the patient's emotional state results in the patient's left verbal posterior processing becoming aligned with the existing right posterior sensory processing or memory activation. In this case the left posterior column activation of the connected frontal columns aligns the left frontal processing with the existing right frontal processing. In both empathy and genuineness, there is less inhibitory input between the frontal lobes in the patient's brain. This is expected to be perceived by the patient as decreased internal conflict.

Improved congruence can result from alterations in action and receptive columns in both hemispheres. The aforementioned act of remaining in anxiety-producing safe situations until emotional distress dissipates allows the right hemisphere to modify its patterns to align with the left hemisphere's appraisal that there is no danger. An additional manner to improve congruence in therapy is providing the left posterior cortex with logical information that right hemisphere receptive processing and action are expected and reasonable.

Unfortunately, there are many things therapists do that can increase hemispheric incongruence experienced by a patient. For example, the all-too-frequently employed question, “Why do you allow yourself to feel that way?” (referring to guilt or some other negative state) immediately registers in the patient's left hemisphere with the interpreter concluding that it should somehow have the ability to control the emotion. Based on the CBM, just the opposite is true in relation to right cortical processing. This is an example of how a brain-based model has the potential to identify which therapists' behaviors may have iatrogenic effects (see Moss [53] for a more detailed discussion).

One ongoing factor identified as relevant to chronic pain patients involves problems in interpersonal relationships. However, to the author's knowledge there has been no discussion of how to address this in the context of individual psychological treatment in chronic pain cases. The CBM provides insight on how this may be accomplished.

### 8.1. Interpersonal relationship behavior patterns

The first description of social neuroscience was provided by Cacioppo and Berntson in 1992 [177]. This rapidly developing field has been facilitated by the pervasive use of imaging technology. A thoughtful review by Adolphs [178] provided insights on the directions and conceptual challenges in this new area, noting the difficulty in identifying the neurophysiological basis of the “social brain” due to there being no brain structure or subpopulation of neurons which operate in isolation. Thus, research was needed to elaborate on the manner in which distributed neuronal representations contribute to social behavior.

In a study involving whole brain fMRI with rhesus monkeys watching 6 types of videos, Silwa and Freiwald [179] focused on patterns associated with social interactions between monkeys. They described the social interactive network (SIN) which involved cortical and subcortical areas, including those of the parieto-frontal mirror neuron system and category selective networks. There was an exclusively SIN (ESIN) that deactivated in all but the social interaction conditions; it included a cluster in ACC and dmPFC; a cluster in vlPFC; area 7a in the inferior parietal lobe; and OFC areas 10o and 14r. They noted the joint characteristics associated with the ESIN bear resemblance to those involved in human theory of mind (ToM) and the DMN. Moreover, they indicated that the human brain regions implicated in ToM and DMN are a plausible homology to those areas in the monkey brain. From a DSM perspective, it is logical to assume that effective treatment related to influential interpersonal negative emotional memories should have components capable of engaging the identified processing areas of the SIN, with specific emphasis on the ESIN. This is based on the DSM proposal that memories involve the same columns as involved with the original processing. In this case, the “gist” memory columns [36] in the medial temporal lobe have their associated action columns in the mPFC. The mPFC's action columns would be those associated with the gist of perceived uncontrollability and personal inadequacy/responsibility related to the SIN.

Genes and culture are the heritable components related to social cognition [180]. An individual's cultural and social context strongly influence the trajectories of innate, biological factors. Bates et al. [181] note that temperament (i.e., a pattern of responses that occurs across multiple situations within a given incentive condition) and environmental interactions determine expression. They discussed an example in relation to novelty distress and harshness. Harshness with low novelty distress children does not appear related to prosocial behavior development, while high novelty distress children fare better with gentle (versus harsh) maternal control. Based on the DSM, both the culturally and socially-based learned components are the result of cortical column processing and memory storage.

As related to social functioning and the SIN, the three most relevant DSM cortical dimensions are internal–external (medial and lateral cortices), action–reception (frontal and posterior cortices), and global–analytic processing (right versus left cortices). The relevance of each will become apparent in the ensuing discussion. Medial cortical areas have reciprocal connections with subcortical structures which relates to Panksepp's [182] discussion of the affective foundations of “core consciousness” and “core self” The “core self” concept fits well with the theorized internal/self-referential columns of the medial cortex that interconnect with the affective systems emphasized by Panksepp. Social interactions also require external stimulus information which is processed and coded in the lateral cortex.

Personality psychology has provided taxonomies of traits in the understanding of individual differences in cognitions, motivation, and behavior tied to relationships. DeYoung and Gray [183] provided a thorough discussion of the emerging subspecialty of personality neuroscience. They discussed the Big Five Model [184] as the most widely used taxonomy and considered it to be a reasonable approach for categorization in personality neuroscience. The five factors of this model are: openness/intellect, conscientiousness, extraversion, neuroticism, and agreeableness. The factors as explained by John and Srivastava [185] are as follows: extraversion involves being energetic, assertive, and talkative; agreeableness involves being trustful, good-natured, and cooperative; conscientiousness involves being dependable, orderly, and responsible; neuroticism involves being easily upset without calmness and neurotic; openness/intellect involves being independent-minded, intellectual, and imaginative. To assist in evaluating the differences and similarities in the information conveyed by the Five Factor Model and the CBM, there will be an analysis after a discussion of the CBM concepts.

As noted by DeYoung and Gray [183], the Five Factors were originally conceptualized as being independent traits at the highest level. Notably, research later showed they have a higher-order factor structure with two metatraits. The first is labeled α, or Stability, and consisted of agreeableness, conscientiousness, and reversed neuroticism. This will later be discussed as relating to Type-G individuals. The second metatrait labeled β, or Plasticity, and is comprised by extraversion and openness/intellect. This metatrait appears to correspond to Type-T individuals. The has been some support for a genetic involvement in the metatraits based on behavior genetic analysis [186]. Furthermore, DeYoung and Gray [183] discuss the accumulating evidence of an association of serotonin with Stability and dopamine with Plasticity. DeYoung [187] elaborated on the role of dopamine to personality related to an entropy model of uncertainty. However, a recent study [188] found only limited support (based on a number of modeling considerations) for the central serotonin connection, and that is true only at the most extreme levels of stability traits.

It was suggested by DeYoung and Gray [183] that structures of the brain may differentially involve these Five Factors. They suggested that functionally relevant brain systems may be correlated with personality traits. Limited support was identified in a structural magnetic resonance imaging (MRI) study [189]. Regardless of some such findings, there are several criticisms of this approach in personality neuroscience. First, the Big Five Model is not based on neuroscientific theory. Second, neither functional nor volumetric neuroimaging of brain areas provides any information on the manner in which brain regions produce any specific patterns of behavior. For example, any correlation between a brain area and a Factor provides no insight as to the social, motivational, affective, or cognitive, aspects involved. Thus, no meaningful information is provided for the purposes of psychotherapy. Accordingly, the finding that someone is high on extraversion which is associated with increased size in the medial orbitofrontal cortex provides no insight in explaining an extravert's behavior patterns to a patient or how to effectively deal with that individual.

In relation to the Big Five factors, the metatraits are explained as related to the Giver/Taker concept. This CBM concept is the one that explains the motivation (i.e., activating positive and deactivating negative emotional states) for behavioral expressions and that emotional valence is determined by the sensory emotional memories in the right cortex. The right vlPFC columnar circuits are posited to determine the behavioral and affective responses of Type-G and Type-T patterns. As will be discussed, the behavioral dimensions of domineering–submissive and socialized–undersocialized will permit an explanation of how the CBM-based relationship behavior patterns directly relate to the Big Five personality traits.

As with the existence of two metatraits in personality theory, there is evidence from neuroscientific research that can support the existence of two distinct patterns related to interpersonal relationship behaviors. Compared to an extravert group, Wang, Chang, Chuang, & Liu [190] found the introvert group showed stronger outflow modulations in the DMN regions operated by the PCC and mPFC. They believed their results were consistent with prior research showing an introversion/extraversion distinction in connectivity between the mPFC and PCC, as well as research supporting the PCC as involved with self-centered cognition that occurs in introversion.

In a study of groups showing selfish versus prosocial actions related to other's social value, Fukuda et al. [191] reported that the left dlPFC and right aIns impacts on the mPFC responses were different between groups related to the value of an offer to others. The offer value was uniquely encoded in the right TPJ for both groups. The mPFC responses were significantly modulated by the right aIns in only the selfish group. In contrast, the left dlPFC modulated mPFC responses in the prosocial group. As will be discussed, the prosocial group is consistent with Type-G individuals who purportedly activate positive feelings by pleasing others and follow rule-governed behavior which necessarily involves the left analytical cortex. Type-T individuals are theorized to activate positive emotions by taking from others based on hedonistic motivations and avoid the use of logically-derived fair rules from the left cortex.

Stable trait-like variants were found in human brain networks based on resting state functional connectivity across the entire cortex [192]. The trait-like variants occur commonly in the lateral frontal cortex and temporoparietal regions, especially in the right hemisphere, as opposed to other cortical regions (appearing to be consistent with the areas identified in the Fakuda et al. [191] study). The variants are often associated with control networks, including the DMN and cingulate-opercular (CO) ones. One group showed stronger correlations with the CO, dorsal attention, and sensorimotor networks. The authors suggested those variants are more strongly associated with control and processing systems. In contrast, the other group exhibited stronger correlations with the DMN. Those individuals in the control and processing group had a higher score in the positive life-experience factor and were lower in a history of drug abuse. The CO group is believed to correspond to the Type-T pattern while the DMN group involves the Type-G pattern, both of which will now be discussed.

### 8.2. Type-T (taker) and Type-G (giver) relationship patterns

Based on the CBM, I [51],[52],[54],[56] hypothesized there are two distinct, but basic, learned behavioral patterns by individuals which lead to activation of positive emotions and deactivation of negative ones in the context of interpersonal relationships. The patterns involve either the taking (Type-T, or taker) or giving (Type-G, or giver) of attention, control, power, and/or material things. This provides an explanation of the basic motivational rules involved in relationship behaviors and is based on both the sensory emotional memories (i.e., how one feels) and action memories (i.e., how one behaves). In addition to hereditary/genetic factors (e.g., temperament) contributing to the development of one pattern over another, each person's own learning history is believed to be equally, if not more, important in pattern development. One's learning history determines what was most effective in avoiding negative and acquiring positive consequences with all influential people within a given individual's early social system. Once the pattern is established, people continue to interact with the current social system in the same basic manner of taking or giving because those earlier emotional memories and overlearned behaviors determine which of behaviors result in positive or negative internal states.

Takers experience positive emotions by taking attention, control, power, and/or things while experiencing negative feelings when having to give, particularly if it is at their own expense and nothing can be obtained. Thus, takers give only when they can obtain or maintain more desirable conditions. If a taker desires attention as her/his priority, there may be a willingness to give up direct control and power. In this example, the individual may be very dependent and whiny, engaging in behaviors that may logically appear maladaptive. In contrast, a taker who most values control relative to attention may actually allow others to receive the public attention provided she/he can maintain control.

Givers experience positive emotions in relationships by giving attention, control, power, and/or things, while having negative feelings when having to take things at another individual's expense. Although being able to behaviorally “take” in some situations, a giver has to develop specific rules as to when that is reasonable. Such rules allow givers to define the conditions under which it is acceptable to take. Notably, the major positive experience for givers is when they spontaneously decide to do something for someone, perceives it was done well, and the recipient shows a genuine appreciation for the act. In contrast, a negative experience involves the giver having to accept something that he/she has typically done, lacks the means to repay what was done, and receives statements from others that induce guilt about accepting the action.

The development of these interpersonal behavior patterns appears logical in the presence of a parallel processing model of the brain in which influential emotional memories are located in the right cortex and the prime directive is to maximize the positive and minimize the negative feelings being experienced. Obviously, the behavioral patterns are based on the motivation for maintaining the behaviors. The sensory emotional memories are what determine the manner in which a person is able to experience positive and negative emotions and lead to the motivation for maintenance of the behavior (i.e., frontal action columns) patterns.

Emotional memories in the right cortex occur early in development, being independent from the left hemisphere verbal-thinking process. Accordingly, it is the right cortical emotional memories that influence all future memories based on the assumption that those determine the experience of positive and negative emotions. Clearly, the most efficient manner of maximizing the positive feelings is by stimulating previously stored positive memories and avoiding activation of previously stored negative memories. After individuals store specific memories associated with either a pattern of taking or giving, it seems logical that this pattern to continue and intensify.

Based on the posterior cortical emotional memories, the right and left PFC develop circuits of columns associated with a person's actions. That individual's frontal action columns lead to behavioral expressions to influence others and their behavioral reactions that activate or deactivate the non-detailed emotional memories of that individual's right posterior hemisphere. Once the PFC actions columnar circuits are formed and lead to subsequent cerebellar control, it is expected that similar new environmental stimulation results in the established behavioral patterns.

It is expected that we each develop patterns of behavior resulting in the most effective means of increasing positive and decreasing negative feelings during our childhood years. Our siblings have done likewise. Therefore, in our own family system, it is expected that we each develop idiosyncratic behavior patterns in the presence of the same basic background. For example, if your older sister learned the most effective manner to increase positive or decrease negative emotions was to be dependent and complaining, it would be difficult for you to maximally capitalize on all available attention, control, and material things by using similar behaviors. Under such conditions, it is expected that you would develop an alternative behavioral pattern, perhaps by being independent and domineering. Regardless of the behavioral patterns that you and others develop, the primary goal is to maximize positive and minimize negative emotions. Thus, it is our family system and our later overall social system that determine what results in positive and avoid negative consequences. Based on the CBM it is our earlier sensory and motor memories that result in our maintaining patterns of interpersonal behavior.

Along these same lines it should not surprising that one's native verbal language (e.g., English) is used throughout our lifetime in social interactions. That applies to past and current relationships, including those with spouse and friends, as well as in school and work relationships. For example, if someone never learned to speak Russian, why would you expect that person to speak Russian in social situations? When considering emotional communications in relationships, is it not equally reasonable to expect that one would always employ that learned during one's developmental years?

Based on the CBM, the most adaptive pattern would be one in which an individual can give and take equally well depending upon its appropriateness in any given situation. This necessarily requires interhemispheric congruence (i.e., both hemispheres are consistent in the analysis and response to all situations). However, each of these conditions is impossible as we live out our developmental years. In explanation, hemispheric congruence requires that we be reared by parents with the ability to communicate consistently congruent emotional and verbal information. As a result, we are presented with an impossible situation. To establish perfect interhemispheric congruence, there must first be people who have perfect interhemispheric congruence. Moreover, if somehow perfect congruence existed, to maintain it an individual would have to be able to filter incongruence communications from others. In other words, interacting with someone who emotionally communicates one thing while verbally expressing conflicting information leads to hemispheric incongruence for that “perfect” person.

Despite the fact that perfect interhemispheric congruence is not humanly possible, it is can be improved, which makes that a reasonable goal in psychological treatment. With improved congruence, a person is expected to give and take more equally well. This theoretically should result in the behavior pattern associated with givers or takers becoming less pronounced. However, the basic pattern remains.

Based on the foregoing discussion, the conclusion is that the patterns tied to being either behavioral type are maladaptive. This is also an important point because patients frequently inquire as to the possibility of one type changing to the other. The answer is clearly “no”. Such a change would be an unreasonable goal even if it were possible. The desired goal for everyone is an ability to give and take equally well in relationships based on what is most adaptive in any given situation. A couple of examples will be helpful in clarifying how each pattern can be maladaptive.

In the first example there is a patient who is a giver who has a severe, chronic pain condition. In the past, he was able to provide the family income and was very active while at home. He lost his ability to do his manual labor job and most physical activities at home because these result in extreme pain. He has guilt when asking his spouse or others to assist him financially or with chores he normally performed. As a result of his giver pattern, he delays in seeking disability benefits, and when denied, he fails to appeal this decision because he feels others believe he should be able to work. He foregoes many things because of his discomfort in approaching agencies, family, or friends for assistance. In this case, this patient's inability to take comfortably restricts adaptive behavior.

A different example involves a Type-T person who develops chronic pain. He previously did little around the house and was manipulative in his job, responding negatively to others' requests that he follow rules. He is complaining, with pain behaviors that are selectively demonstrated to achieve his goals, whether it is interpersonal manipulation or attempting to show his level of disability to get monetary benefits. However, when wanting to do something he desires, he does so often without pain behaviors being demonstrated. He readily asks for assistance from agencies, family, and friends, complaining that no one knows the extreme level of his multiple life problems and saying no one cares regardless of how much he has been provided.

As one first looks at these behavioral patterns, there is usually a tendency to see the “trees” (i.e., specific behaviors) and not the “forest” (i.e., the emotional motivation for the behaviors). In other words, it may be difficult to reconcile the fact that the same basic type of individual can show extremely different behaviors. Similarly, it may be difficult to accept the fact that two individuals demonstrating similar behaviors actually reflect different basic types.

For example, one taker can be highly successful, socially adept, and regarded by the public as a philanthropist. In contrast, a different taker may be complaining, obnoxious, and extremely dependent and play out the role of a victim in relationships. In reference givers, one may have strong beliefs and appear opinionated and demanding, while another may be compliant, submissive, and barely noticeable.

It is proposed that each basic type can vary along two dimensions which assists in recognizing the patterns. In relation to psychotherapy the basic patterns are the focus of attention, not the subdivisions of the basic types. Due to the basic patterns being so pronounced, the distinction of Type-G versus Type-T is best viewed as a dichotomy. The greater the hemispheric incongruence, the more pronounced the patterns become. However, the two dimensions are viewed as continuums, and as such, are not mutually exclusive and independent.

The two behavioral dimensions for each basic type are (a) domineering–submissive and (b) socialized–undersocialized Socialization involves the extent to which a person employs socially acceptable behaviors in the process of activating positive emotions and deactivating negative ones in relationships. Dominance refers to how much one maintains relationship control.

With the dimensions in mind, it is possible to formulate prototypes of individuals falling at the extreme end of each continuum. A dominant/socialized giver is an ethical, strong-willed individual who has clearly defined rules. She/He would be competent and conscientious in work situations and, if in a supervisory role, would attempt to be loyal and fair toward both employees and the company. She/He is willing to work with others and not expect anyone to do what she/he is not willing to do. Such a person is expected to be self-sufficient and to assume significant responsibility in the home, community, and/or office, while not be overly attention seeking due to feeling uncomfortable with too much public attention. When recognized for her/his contributions and achievements, there is enjoyment in having the recognition but a tendency to also give credit to others. In reality, she/he would feel more pleasure having people say nice things about him/her rather than to him/her. This is based on the expectation that direct compliments result in a feeling that she/he is being self-serving (e.g., conceited) by openly accepting the compliment.

A dominant/undersocialized giver should only rarely be seen. The pattern is actually the same as the socialized dominant type, with the exception that the rules being followed are, from a societal point of view, inappropriate and possibly illegal. An example of such an individual is the stereotypical Mafia godfather who is loyal and rule-governed, but engages in illegal activities.

The submissive/socialized giver consistently attempts to please everyone and avoids interpersonal conflict. This individual is perceived by others as a generally nice person. Though not necessarily volunteering to do much, this type has an inability to comfortably refuse to comply with requests of others, regardless of the inconvenience. She/he is expected to do a competent job when agreeing to do something, but avoid too much responsibility due to fears of conflict and feeling that others could do a better job. As a spouse, this person is frustrating due to his/her avoiding any conflict, often “pulling into a shell.” He/She tends to leave and avoid any conflict situations.

Submissive/undersocialized givers would be escapists, typically involved in socially unacceptable behavior that does not directly harm others. They would often be invisible in society. One example is a person who quietly abuses drugs or alcohol. They would tend to be “homebodies” or hermits, while if homeless, they would be aloof and isolated. Such individuals would only marginally function in society and are not expected to seek attention or help.

A dominant/socialized taker has the strong need to control others in a direct fashion, but does this in ways that those not close to him/her perceive as socially acceptable. They are expected to be outgoing and socially adept, particularly during first social encounters. Their charm and charisma are shown if they desire something in a situation. Often, they do things for public recognition and relish praise from others. In contrast, those close to them are aware of very different behavior when others are not present. They are demanding and temperamental, often being verbally abusive or cold when not immediately getting their way. They lack empathy, often be callous, in their one-on-one interactions with those in close relationships.

A dominant/undersocialized taker is the most dangerous type. They strive to have total control in relationships, often going to extreme levels in pursuit of control. She/He often loses jobs due to an unwillingness to follow directions from others, and many engage in illegal activities. They abuse family members verbally, physically, and sexually. After openly expressing anger and turning cold to achieve immediate gratification, he/she may actually be apologetic or otherwise nice for a brief time if that is needed to gain what is desired. However, the pattern soon repeats. If intoxicated, they are often described as “mean drunks” and may engage in physical fights. They have no real loyalties to anyone and will use friend and stranger alike. In a relationship in which they have mutually (i.e., both themselves and the partner) perceived power, they escalate emotional, physical, and, sometimes, sexual abuse over time. If the partner is perceived as the one with ultimate power and control, they often escalate their own aggressive behavior to the point that the partner either walks away or attacks.

The submissive/socialized taker primarily seeks attention. They may be seen as a giver because they are often involved in many public activities. Notably, they make their presence known to others and, in social gatherings, are frequently considered the “life of the party.” In their close relationships, they often cause others to feel guilty because they play the role of the martyr. Despite doing a great deal for others publicly, it is those close to them who must handle the more mundane day-to-day matters of the household.

The submissive/undersocialized takers typically receive attention in socially questionable or sometimes inappropriate ways. They are often the people threatening suicide or self-mutilating, and are often placed on a number of medications when treated psychiatrically because nothing seems to work. Some have a number of psychiatric diagnoses and/or claim to have physical problems. Some often say they are the victim and martyr, while many will go to great lengths to play out the role.

Only a brief discussion of each type has been presented. The hope is that the information is sufficient to convince clinicians and researchers that these types can theoretically exist. If valid, the ramifications would be widespread in the many areas of psychology. Consistent with cognitive-behavioral approaches, the descriptions provide schemas that explain the actions of others in a patient's problematic relationships which can assist in externalizing which decreases the perceptions of personal responsibility/inadequacy tied to past memories. An important aspect is the information assists patients in forming realistic expectations of their current relationships (e.g., marriage, divorce, work) and how to most effectively deal with those individuals. The information is logically-derived, being based on the described motivation for the behavioral patterns of each type. The interested reader can find a more detail discussion of how the information is used in treatment in prior professional [54],[55] and self-help [193] publications.

In relation to the Five Factor Model, it is expected that Type-T individuals as a group are expected to be low in conscientiousness and high in extraversion. Taking in relationships is expected to involve an active behavior pattern with disregard of rules to maximize gains. The submissive/socialized and dominant/socialized individuals would show greater intellect/openness, particularly as related to being independent-minded and imaginative. Those within the submissive/socialized category are predictably higher in agreeableness. Individuals who are classified as submissive/undersocialized and domineering/undersocialized are expected to be lower in agreeableness and higher in neuroticism based on the tendency to get easily upset and distressed, while failing to be trustworthy and cooperative.

In contrast to taker patterns, Type-G individuals are as a group expected to be high in agreeableness and conscientiousness. They desire for others to see them as good and not bad, resulting in attempts to please others and follow rules. Individuals who have a submissive Type-G pattern are predicted to be higher in neuroticism and lower in extraversion. That is based on expected conflict avoidance and a tendency to be uncomfortable as the center of attention, while also having sensitivity to criticism and being easily hurt. People who are domineering Type-G would be higher in intellect/openness, specifically as related to being independent-minded and intellectual.

Based on a CBM view, Type-G individuals are those who may have relatively higher serotonergic activity while Type-T individuals would be higher in dopaminergic activity. If this correlation were to prove accurate, it is unclear as to whether the neurotransmitter differences contribute to behavioral pattern development or are the result of those behavioral patterns, or both.

A greater discussion of how the CBM relates to various psychotherapy approaches in dealing with ongoing factors (e.g., relaxation approaches) has been provided elsewhere [37],[54]. At this point, the discussion will turn to another area that has not been discussed in pain treatment.

## 9. Negative emotional memories

The theoretical aspects tied to negative emotional memories based on the clinical biopsychological model and supporting studies have been previously described [53]. Only the most salient points will be included in the current discussion. First, it is clear that all humans experience a number of situations resulting in negative emotions, but these do not necessarily lead to memories contributing to psychological problems. Therefore, there must be some aspects in relation to the situations which account for such individual differences.

The sensory aspects of negative emotions are stored in the posterior cortical lobes and the associated actions are stored in the frontal lobes. It has been proposed [37],[52]–[54] that the two major situational factors tied to detrimental impact of all negative emotional memories are perceptions, or feelings, of (a) loss of control and (b) personal inadequacy or responsibility. These two aspects have been similarly noted in relation to traumatic memories by Foa and Rothbaum [194] where they describe patients' beliefs that the world is totally dangerous and they themselves are completely incompetent. In a meta-analytic review of laboratory studies of stress, Dickerson and Kemeny [195] concluded that lack of control and social-evaluation were associated with the most intense and longest duration of arousal. Tasks containing both uncontrollable and social-evaluative elements were associated with the largest cortisol and adrenocorticotropin hormone changes and took the longest time to recover. Thus, there is evidence to support the importance of these factors. The CBM proposes that whenever a current uncontrollable situation occurs, past negative emotional memories associated with perceived uncontrollability can be reactivated. If those prior memories were associated with both perceived uncontrollability and feelings of personal inadequacy or responsibility, current situations without a social-evaluative component may still result in the inadequacy feelings via past negative memory reactivation that had that aspect.

It has been shown that controllability related to pain is a factor determining differential activity in the brain. Brascher, Becker, Hoeppli, and Schweinhardt [196] assessed the role of pain perception and controllability with non-pain subjects using a within-group design. Controllable pain was associated with increased negative connectivity of the dlPFC with aIns and thalamus. The authors suggested this supports the importance of the dlPFC in pain-inhibitory effects of controllability. Uncontrollable pain (which resulted in increased sensitization based on pain ratings) led to increased connectivity of the mPFC with the aIns, ACC, and inferior parietal lobe. There was a non-significant stronger coupling between the amygdala and aIns in uncontrollable pain. The authors believed the results support the role of mPFC as a driver in pain sensitization. Based on the DSM, an available external (lateral cortex) method to control pain requires dlPFC (external action columns) attention and motor response initiation. With attention being externally focused, there is less connectivity to the aIns and thalamus because those areas are not online based on the task at hand. In the uncontrollable pain condition, there is no external stimulus requiring dlPFC attention involvement. It is expected that focus is on somatosensory (inferior parietal) and interoceptive (aIns) pain processing involving intentional volition (ACC) related to the mPFC (internal action columns).

As has been previously discussed, the mPFC has been is involved in the transition from acute to chronic pain. In pain patients with a history of emotional resilience, there is expected to be an internal perception of controllability (e.g., ability to cope) related to most life situations, including pain. Patients lacking such emotional resilience are expected to perceive lessened internal controllability. If accurate, then columnar circuitry within the mPFC is an area of importance in psychological treatments related to enhancing resilience in chronic pain patients. Therefore, it is proposed that the self-referential aspects of perceived uncontrollability and personal inadequacy/responsibility action columns are in the mPFC, most notably in the right hemisphere. However, the lateral frontal cortex is bilaterally involved with the processing of external input. Therefore, treatment that alters mPFC and lPFC are important in both the right and left hemispheres to increase resilience.

Earlier traumatic and non-traumatic (particularly in significant relationships) negative events appear to be very influential in leading to psychological disorders associated with a current trauma and other negative emotional situations [53]. In this case, the memories which are formed prior to a recent traumatic situation are those leading to the maladaptive patient beliefs described by Foa and Rothbaum [194]. There are physical health effects of childhood negative emotional memories beyond those previously discussed as related to chronic pain. Research has demonstrated early adversity impacts susceptibility to conditions involving pro-inflammatory processes, including heart disease [197], in addition to fatigue in chronic fatigue syndrome [198] and breast cancer [199]. Based on the CBM, the right cortical memories are the most influential in leading to maladaptive emotional states and behavioral reactions, yet the most difficult to access in psychotherapy. As previously noted, all psychotherapy necessarily involves the left cortical verbal-thinking region which does not have direct access and control over right posterior processing and memories. The majority of chronic pain patients I treated over the past 39 years had prior influential negative memories, most often involving past and current relationships. Although most people have some degree of feelings of lost control when experiencing a traumatic event or disabling pain, those with prior relationship negative emotional memories are much more prone to experience the personal inadequacy aspect. If only the loss of control aspect is perceived tied to a traumatic event, this will likely be responsive to exposure-based therapies [53],[54]. However, in relation to negative emotional relationship memories, repeated detailed factual discussions of such memories in psychotherapy leads to little overall improvement and may actually increase distress. Prior to discussing the treatment of relationship negative emotional memories, a discussion of how adaptive functioning tied to memory storage and cortical processing theoretically occurs is warranted.

Only a minority of individuals exposed to trauma experience persistent psychological problems. For example, Housley and Beutler [200] looked at combined results of three reports and found a 12 month-prevalence rate for posttraumatic stress disorder and acute stress disorder of 12% in the general population. Thus, the majority of people somehow have the resilience to handle trauma. In relation to the CBM, there are several factors which can explain such resilience.

In the right hemisphere, resilient individuals likely have few posterior cortical receptive negative emotional memories stored with the loss of control and personal inadequacy/responsibility factors. As such, the posterior cortical sensory negative emotional memories tied to past situations or events are necessarily linked to associated lateral and medial frontal action columns in which the situations were adequately managed or controlled. In the event of a traumatic situation, the similarity of the emotions activates the posterior columns which in turn activate the frontal columns tied to past successful coping behavior. In relation to left frontal cortical memories, successfully managing the past negative emotional situations was likely a result of the logical analysis and response mediated by the verbal interpreter. There is also a strong likelihood that resilient individuals have the verbal-thinking ability both to label accurately and accept the reasonable nature of negative emotions. The mPFC action columns associated with the medial temporal lobe receptive columns are expected to be those associated with perceptions (i.e., the gist) of personal control and adequacy. In total, these cortical factors result in feelings and thoughts that one is capable of effectively coping with any situation, with the verbal interpreter often initiating action to address immediate needs.

In maladaptive functioning, the frontal columns of both hemispheres lack a history of successful and socially appropriate personal control behaviors. In this case, the only right hemisphere generalized behavioral responses of the action columns are to attack, freeze, escape, or avoid. If this is the case, the left verbal interpreter lacked a means of effectively dealing with similar situations in the past and the right mPFC gist memory is one of personal inadequacy and lack of control. Based on this theoretical formulation, the right hemisphere is most likely to assume control over responses in those lacking a history of effective verbal-thinking behaviors.

Given its lack of extensive verbal abilities, the externally-directed attack response of the right vlPFC response is characterized by verbally and/or physically aggressive behaviors. If this behavior is observed, there must be a history of at least limited success. However, despite possible immediate success in terminating a negative stimulus, there are obvious longer-term social consequences to such behavior. Although there may be less chance for a loss of control feeling with aggressive behavior, there is unlikely to be a feeling of personal adequacy. The other action responses of freeze, escape, and avoid lead to both loss of control and feelings of personal inadequacy. As already noted, “personality” is considered to be the right frontal action column-related behaviors based on the receptive columns (and their associated positive or negative emotional response) which activate in response to relationship behaviors of others. Therefore, “personality disorders” can be defined the same with the only addition being that the relatively stable pattern of behavior is considered maladaptive. Overly manipulative (i.e., aggressive or controlling) right action column behaviors are characteristic of antisocial, narcissistic, and borderline patterns. The freeze, escape, and avoid right action column behaviors are characteristic of avoidant or dependent patterns.

The importance of engaging emotion directly in psychotherapy has been noted by Greenberg [201]. His approach, called emotion-focused therapy (EFT), employs experiential techniques which primarily impact the right cortex as based on the CBM. As I read case descriptions of EFT and other experiential procedures, as well as Eye Movement and Desensitization Reprocessing [202] approach, there is a characteristic transition within-session for successful clients. Initial anxiety transitions to anger (often with expression) followed by a feeling of self-sadness and relief. However, EFT and other experiential approaches lack a structured process to identify potential influential memories to be targeted in treatment and a structured manner to elicit the therapeutic within-session emotional transitions. I have [37],[51]–[54] advocated for the use of a structured assessment to identify all potentially relevant relationships (e.g., spouse, parents, work supervisor, etc.) because each can contribute to a patient's current emotional functioning. Once identified I also support the use of a structured treatment approach (i.e., emotional restructuring, or ER) to address each target relationship. Based on the CBM, and in relation to the most effective ways to access the right cortex, the use of experiential (e.g., empty chair technique, role play/reversal) and imagery (e.g., directed imagery, metaphor) techniques are essential.

ER [37],[53],[54] has been used clinically in the treatment of problematic memories for any given past or present relationship. This structured treatment involves six steps conducted in one to two one-hour sessions. For the interested reader, all details on the procedure are outlined in a treatment manual [54]. It is important to note that the approach has not been subjected to randomized clinical trials because it was developed in a private practice setting with no available means to conduct such research. The following provides a description of each step and the theorized brain effects based on the DSM.

### 9.1. Negative emotional memory recall

The first phase involves having the patient recall specific negative events/situations that occurred within the targeted relationship being addressed. In the process of recall and at any other times in the session the patient makes verbal responses, the verbal interpreter (left vlPFC) is involved. In emotional expressions, the right vlPFC (i.e., the emotional interpreter previously discussed as involved with categorizing and expressing emotions) is involved throughout the session. When the patient listens to the therapist's verbalizations, her/his left posterior cortical areas are engaged, while therapist emotional expressions involve the patient's right posterior cortex. The major effect of recall is reactivating autobiographical negative emotional sensory memories housed in the right posterior lateral and medial cortex. This is expected to result in amygdala activation and associated sympathetic arousal. There is expected mPFC activation as well, based on connectivity with the amygdala, Ins, and cingulate cortex. The patient's perceived emotion is typically increasing anxiety during the process of discussing the emotionally-charged information. The patient is asked to identify one or more past memories associated with significant anger toward the target individual to use during the anger-release imagery phase.

An important point based on the CBM relates to discontinuing a session in which there is only discussion about the past negative situations/events. In that instance it is expected that the patient will leave the therapy situation feeling increased distress. The theoretical basis is that associated memories reactivate mPFC feelings of personal inadequacy or responsibility with nothing therapeutic having occurred to mitigate those perceived feelings. Additionally, the patient may actually form new, negative memories of the therapy room and therapist which can decrease the patient's motivation to return for additional sessions due to anticipatory anxiety associated with the negative memories of the former session. For many patients with influential negative emotional memories, it appears the default response for many patients with influential negative memories is to avoid memory reactivation because there has been no reduction in the impact of those memories when they were discussed in the past.

### 9.2. Interpersonal relationship behavior description

The next phase involves presenting a behavioral description of the previously discussed giver or taker pattern. Obviously the verbally-presented information engages the patient's left temporal and temporoparietal areas required for language comprehension. The posterior left columns may activate right posterior columns in two ways. First, verbal information may result in right posterior cortically-mediated visualization. A second possibility involves the description's explanation for past events and behaviors related to the target individual. During the presentation as to why the target individual behaved in the described manner, in conjunction with the patient's spontaneous recall of additional past interactions in which those behaviors actually occurred, the patient's verbal interpreter gains powerful new insights. The verbal interpreter applies the information to the additional situations which results in right hemisphere visualization. The verbal interpreter can accept the logical information with acceptance of the reasonable emotional reactions emanating from the right hemisphere, leading to improved interhemispheric congruence and lessened internal conflict. This is often when the first perception of anger (right Ins, cingulate cortex, amygdala, mPFC, and vlPFC) occurs (i.e., anger is conceived as an expressive emotion related to action columnar circuitry). The CBM explanation is that there are new right frontal columns associated with the new posterior cortical visual images (recall that with each new posterior column, a new frontal column forms) allowing the expression of right vlPFC-based emotions.

### 9.3. Role reversal/role play

During the role reversal there is a brief interaction in which the patient is instructed to assume the role of the target individual while “playing by his/her rules” which are described. Following the detailed descriptions, the basic rules (left vlPFC) that guide behaviors being shown are greatly simplified for the role reversal. If the target individual is a taker, the basic rule is “I win; I get my way no matter what I have to say or do.” In the case of a giver, the rule is “I want to be a good person and avoid feeling like the bad guy.” The therapist then assumes the role of the patient and initiates the interaction. In situations that the patient has difficulty assuming the role of the target individual, the therapist can assume the target individual's role to model the verbal and emotional behavior. The role play/reversal process typically lasts less than a minute in most cases.

The verbal interpreter is involved in a unique manner during the role reversal. It is provided a new set of rules which are attributed to the target individual. Simultaneously, the verbal interpreter has access to memories of past verbal exchanges from which to draw and is to use those emotional verbalizations (i.e., simply repeating what the target individual said in past situations) while the patient is pretending to be the other person. There are also a large number of situational memories accessible to the right frontal cortex which enable the mimicking of voice intonations and inflections used by the target individual. Patients who use both the non-verbal voice expressions and the verbalizations employed by the target individual directly activate the left and right hemispheres' frontal lateral and medial action columns. Patients who effectively engage during the role reversal report a major increase in perceived anger (right Ins, cingulate cortex, amygdala, mPFC, and vlPFC). Despite the fact that many patients already concluded at a verbal, logical level (left vlPFC) that the target individual was responsible/at fault, for most patients it is the first experience with the right hemisphere's deep (i.e., with conviction) emotional response.

### 9.4. Imagery for anger release and self-nurturance

Imagery is the component that results in the most rapid and pronounced emotional changes in the ER session. The patient closes her/his eyes and visualizes the therapist-described scene which involves an interaction with the target individual. As the imagined interaction approaches the end, the patient is described as either by physically attacking (if the person was physically or verbally abusive) or deserting/neglecting (if the person failed to provide protection or assistance) the target individual. Immediately after the anger imagery, the description changes to a funeral scene in which the deceased target individual is initially believed to be dead and in a coffin. However, when she/he actually looks inside the coffin the patient sees herself/himself as the deceased (at the age where the most damaging events occurred). As the described scene progresses there is a dialogue of the current age patient with her/his younger self in which it is acknowledged that self-blame for the problem interactions had been erroneously given. At the conclusion, the younger victim returns to life and both the younger self and current age patient give each other affirming statements and hugs.

The activation of non-verbal sensory emotional memories within the right cortex most likely results from the left posterior cortical areas which are activated by the therapist's verbalizations. With the activation of the right posterior columns there is activation of corresponding right dlPFC and vlPFC, as well as mPFC, columns. It is also expected that the amygdala becomes involved via the right posterior cortex. When the anger expression imagery is presented, the new right posterior column circuitry results in the formation of new right frontal action column circuitry. This may result in mPFC-mediated perceived control and personal adequacy. The unexpected shift to the funeral scene takes advantage what appears to be a frequently present right cortically-based feeling for many patients; that feeling is that by defending oneself there will be the loss of a desired relationship and being viewed as a bad person by others. With an acknowledgement that the patient is the actual victim there is an alleviation of such fears with an immediate ability to self-nurture. Throughout imagery, the right and left vlPFC are presented with emotional and verbal information, of which the most important is likely related to the self-nurturing communications. Improved interhemispheric congruence is expected because the verbal interpreter is labeling and accepting the emotional pain associated with the right sensory memories as reasonable and normal. There is both relief (i.e., negative reinforcement by terminating a negative stimulus and leading to mesolimbic dopaminergic involvement) and sadness for self, with those feelings expected to involve bilateral mPFC, OFC, amygdala, Ins, cingulate cortex, PAG, and NAc.

### 9.5. Origins of the relationship pattern description

This phase involves frequent images that includes a description of the target individual whose “inner child” is trapped in a transparent hard-inner shell and unable to grow beyond the shell. The detrimental behaviors from the target individual are explained via the image of his/her attempting to fill an emptiness that can never be filled. Additional imagery is used to facilitate an understanding that the target individual was a victim of her/his own past which resulted in the entrapment of the child. The last part invokes images to support the fact that the target individual was not withholding desired positive and compassionate behaviors, but was unable to give these because they were nonexistent.

The imagery involves the same brain mechanisms described in the previous section. New verbal schemas are formed (i.e., action column circuitry) within the verbal interpreter based on the left posterior receptive columns activated by the therapist's verbalizations). Additionally, new right receptive and action columns form during the visualization. For most patients, pity (i.e., sadness for other) becomes the predominant emotion at this point.

### 9.6. Role played forgiveness sequence

The final phase involves an expression of sympathy for the target individual. During role play the patient is directed to verbalize the emotional damage inflicted on her/him, being followed by an acknowledgement that the target individual was not withholding what was desired and needed, but simply lacked the ability to give to the patient what did not exist. This is based on the fact that the target individual could not be expected to provide what he/she had never experienced. A statement of forgiveness is the final aspect. A frequent observation is that a patient has difficulty overriding the long-standing emotional and verbal (right and left frontal columns) schemas that the target individual really was capable of demonstrating the desired behaviors, leading to reticence in admitting the person did not have the ability to act in a healthier fashion. With gentle verbal reasoning, the therapist can guide patient into the recognition that acknowledging the target individual's shortcomings is not condoning, but simply a reality-based statement of the limits of the person's emotional language ability. This allows the patient to feel (mPFC) there was nothing about her or her (e.g., “I am unlovable”) that caused the target individual's unhealthy reactions. Instead, the problems were a function of that individual's shortcomings (e.g., “you were incapable of loving me in the manner that I needed”).

The process of the ER session involves both the left and right posterior and frontal cortices extensively. The purported areas of involvement include those described as related to the previously discussed SIN [179]. Conceptually, the patient has formed new medial and lateral frontal action columns bilaterally associated with perceptions of control and personal adequacy. Simultaneously there are feelings of self-compassion and other-compassion. The emotional state present at the conclusion of the session is difficult for the patient to describe because it has never been experienced in the past. It involves a mixture of profound relief and cleansing sadness. There are both left frontal (verbal understanding, or new verbal schema) and right frontal (emotional understanding, or new emotional schema) action columns.

In relation to negative emotional memory treatment, the applied theoretical model has value in evaluating the other procedures currently employed in addressing negative emotional memories and the degree to which each can effectively engage the frontal lobes bilaterally. This can facilitate communication among therapists with varying orientations since there is a common ground to understand how the brain is theoretically being affected.

## 10. Positive emotional memories

Failure to activate positive emotional memories leads to predictable patterns of reactions. I [52],[54],[56] proposed a way to conceptualize the reactions for the client based on opponent-process theory [203]. The opponent-process concept originated as related to color vision [204]. In 1973, Solomon and Corbit [205] applied the concept to acquired motivational states in addictions, and they further elaborated how this applies to all acquired motives a year later [206]. The basis of the theory is simple: When an affective state (whether positive or negative) occurs in response to a stimulus, a “slave” opponent affective state activates and gradually strengthens if the stimulus continues to be present. With the presence of both the original affective state and the gradually intensifying opponent affective state, the perceived level of the original emotion reduces over time. It is similar to adding a positive and a negative number together in which the sum is always trending toward zero (i.e., more emotionally neutral). The larger the initial intensity of affect in conjunction with the duration and number of exposures of the stimulus, then the greater the opponent affective state if the stimulus is terminated. Importantly, the opponent state will deactivate over time provided there is no reinstatement of the original stimulus.

Solomon [203] posited that such a process is of biological significance because there are both physical and psychological costs associated with affective states. Accordingly, there is a reduction in the costs when the affect lessens. I [54],[56] proposed that the opponent-process has survival value in a different way. It encourages the person to maintain engagement with the environment by preventing anything from becoming so highly positive or negative that it would interfere with that engagement. For survival to occur all biological needs must be met which necessitates environmental engagement.

Solomon [203] commented on the theory's consistency in predicting experimental outcomes in that every experiment designed to test the model supported its validity. He expressed the belief that the theory had potential applications in areas such as psychosomatic medicine and social philosophy. However, to my knowledge this theory has only been applied clinically to addictions.

### 10.1. Loss and depression

If one applies the opponent-process theory in relation to losses of desirable situations experienced by patients, it is quite sensible as to why depressive symptoms inevitably occur. Based on opponent-process theory, the various losses that occur for many, if not most, chronic pain patients are expected to result in depressive symptoms as a result of positive affective state deactivations associated with each loss. The greater the intensity and the longer the duration of a desirable situation, the more extreme will be the opponent affective state with the loss of that situation. If there is a minor loss (e.g., taking an enjoyable week-long vacation) then only a mild sadness is expected upon returning to a normal routine. However, if there is a major loss (e.g., a primarily positive marriage that lasted 20 years) there is a much greater level of dysphoria. Although this concept appears logical and simple, there has been an absence of application of opponent-process theory to depression in which loss is a major component. There are several factors that contribute to a lack of recognition of the importance of loss-related depression.

A major factor is that elimination of undesirable emotional states regardless of their causes is a primary goal for many professionals who treat depression. Obviously, prescription medication is the most common way depression is treated regardless of causal factors. However, it also appears that many psychotherapists fail to use a conceptual model based on causal factors in their assessment and treatment of patients.

Another aspect is that loss is only one factor that contributes to depression. As has been discussed, current situational factors and negative emotional memories are often involved. The current factors are expected to be the ones most often addressed in treatment with chronic pain patients. This may be the result of the clinician being unable to reinstate losses and a belief that there is no specific way to neutralize detrimental emotional memories.

Another aspect is that losses are often not initially seen as permanent. This may lead to one having no perception that losses contribute to later developing depressive symptoms. For many patients who develop chronic pain, the original injury or illness results in immediate losses, but these are viewed as temporary in nature. Those patients believe that with effective treatment the pain will be eliminated. In disabling chronic pain conditions, the losses can become permanent. If the opponent-process theory is applied to this example, the cognitive expectations that the problem will eventually resolve and there can be a return to one's normal lifestyle offset the depressive symptoms associated with one's being unable to engage in activities. Only a mild dysphoric state is expected under such circumstances. However, when a patient reaches the conclusion that her/his condition is permanent, the ensuing depressive state is expected to be much more extreme. Similarly, expectations can influence the opponent-process. The fact there has been no actual loss can influence the opponent-process despite loss being anticipated. For example, when a person anticipates the death of a close loved one, the anticipatory grief is not as severe as the grief experienced when the loved one dies.

If this aspect is accurate, it then follows that a patient being aware there is no hope of recovery from a non-life-threatening physical condition should experience more extreme depression immediately and complete the grieving process much sooner than someone who is told the condition may only be temporary. Opponent-process theory predicts that several months afterwards, individuals with permanent loss would be less despondent than those whose condition is still present, but considered temporary. This is the result of the eventual weakening of the negative opponent-process state because the physical condition was accepted as permanent. In contrast, those patients who view the condition as temporary maintain the positive affective state based on hope the condition will resolve despite its actually being present. In that case, there is a milder dysphoric state expected. Consistent with this prediction, Smith et al. [207] compared reversible versus irreversible colostomy patients six months following release from the hospital. Ratings of life satisfaction and overall quality of life was better for irreversible patients. The authors speculated that awareness of the temporary nature can interfere with adaptation and result in the paradoxical situation that people who are doing better objectively are subjectively worse. This aspect is common with chronic pain patients who believe there is a possible cure for the pain despite the reality being there is often nothing that will eliminate it.

Another aspect that impacts the perception of a temporal association between losses and depressive symptoms for chronic pain patients is that there tend to be multiple losses across a prolonged time. The pain is expected to interfere with that person being able to comfortably resume normal activities, often leading to losses related to one's job, financial independence, functional independence, relationships, and ability to do physical recreational activities. With the loss of those prior activities, depressive symptoms are expected based on opponent-process theory. With each realized loss, there would be worsening of depressive symptoms associated with that loss. As an example of a primary condition leading to a different kind of loss, Kirchner and Lara [208] provided data that support the manner in which a primary physical condition can result in other areas of loss. For a sample of 65 multiple sclerosis patients the loss of social functioning was more influential in depression than was loss of physical functioning.

A final factor is that losses may not be recognized because they occur within the context of positive life changes or events. For example, when one accepts a new and desired job, there can be an associated loss of close relationships. Even when one achieves a major goal, such as an educational degree, the goal has been lost and some despondency is expected. In a postpartum depression longitudinal study [209], 24 women were interviewed at one, three, and six months after the birth. The authors described paradoxical findings in that the women had positive feelings tied to motherhood, yet they were unhappy because of associated losses. Those losses were related to appearance, autonomy and time, femininity and sexuality, and occupational identity. The author suggested that had the women been taken seriously and encouraged to grieve the losses, it was likely they and their social support network would have interpreted the grief as a healthy process as opposed to a pathological response. This is consistent with the proposed CBM treatment approach [54],[56].

There is another aspect that can be helpful to some patients. If there is a primarily negative life situation, its termination will predictably lead to a positive affective opponent-process (i.e., relief, elation). Although not directly leading to depressive symptoms, the lack of a grief reaction in certain situations can lead to feelings of guilt. With an increase in guilt, there can be increased depression. This can occur when a primarily negative relationship with a parent or spouse ends due to that individual's death. The opponent-process theory predicts there should be a positive emotional state, such as happiness and/or relief. A patient who accepts this is a normal reaction as opposed to being pathological can avoid anxiety and guilt.

A theory of depression based on the loss of reinforcing activities was proposed by Lewinsohn [210] and may be the one closest to the opponent-process theory. Lewinsohn proposed that a low rate of response-contingent positive reinforcement (RCPR) causes and maintains depression. The lower-level antecedents to a low RCPR included: skill deficiencies (e.g., social skills) that limit the elicitation of environmental rewards; a deficiency of the individual to enjoy potentially rewarding events; and an environment lacking desirable reinforcers. The lack of desirable reinforcers was considered to result from personal losses, socioeconomic limitations, and/or major life changes.

The are several differences in the currently proposed opponent-process theory of loss-related depression from that by Lewinshon. His theory views the lack of RCPR as causing depression while the current theory does not. In actuality, Lewinsohn's posited reward deficiency can be explained by opponent-process theory in that the inability to experience positive reactions in response to previously reinforcing activities is a function of the strong negative opponent emotional state. As opposed to being considered a deficiency of the individual, it is a considered to be a normal emotional reaction to a significant loss. Additionally, the CBM proposes the internal opponent-process affective state is what creates the depressive symptoms as opposed to a lack of available reinforcement. With the loss of a significant relationship there are not necessarily limitations on the patient's access to social events, movies, video games, etc.; however, the patient is expected to have reduced interest and enjoyment associated with these activities. The result is that a patient may decide to forego these for a period of time.

At this point there can only be speculation as to the exact neurophysiological basis of the opponent-process. The CBM viewpoint [54],[56] is that there is an inability to reactivate right posterior cortical positive emotional memories of the lost stimuli that leads to the opponent-process depression. The positive lateral and medial emotional cortical memory circuits connect to the basal ganglia, amygdala, and mesolimbic dopaminergic system.

One possible mechanism involves the interactions of the septal regions and amygdalae (cf., Grossberg & Schmajuk [211]). Another possibility has to do with the ventral tegmental area which has connections with the amygdala and NAc. Hollerman & Schultz [212] discussed dopamine signaling in relation to reward exceeding or falling short of expectations. A strong dopamine signal in the ventral tegmental area occurs with unexpected reward. There is a signal reduction associated with repeated reward presentations. In contrast, there is a reduced dopamine signal with omission of an expected reward. The authors proposed that expectancy is the factor leading to decreased or increased signaling; an alternative explanation is that the signal change occurs as a result (or possibly partial cause) of the affective opponent-process. Mink [213] notes that the ventral striatum receives input from olfactory and limbic cortex, as well as the amygdala and hippocampus. There are reciprocal connections between the ventral striatum (including NAc) and the ventral tegmental area. The amygdala and ventral striatum provide input to the ventral pallidum which projects to the dorsomedial nucleus of the thalamus which, in turn, projects back to limbic cortex. Mink suggests that the inhibitory output of the ventral pallidum may act to suppress or select potentially competing limbic mechanisms. Functions of those limbic mechanisms appear consistent with an opponent-process.

In relation to cortical memories tied to significant loss, there are numerous emotional memories involving the external (lateral cortex) and internal (medial cortex) sensory columns. Specific objects and places memories involve the sequential (ventral cortex) information stream while spatial and contextual memories involve the simultaneous (dorsal cortex) information stream. In the presence of association memories, the medial temporal cortex and associated mPFC columns are involved. The loss of positive affect with an ongoing positive stimulus would necessarily involve output from external and internal action columns, though the internal columns (e.g., mPFC, OFC) are likely those projecting to the aforementioned ventral basal ganglia system.

Loss can be considered extinction of prior association memories tied to the previously rewarding stimulus and involve the previously discussed dopaminergic PFC and basal ganglia processes. In this case, the various stimuli (e.g., locations, time of day) previously associated with the absent person, object, or situation are experienced. Theoretically, extinction refers to the weakening of synaptic connections that allowed the formation of the medial temporal cortical columns (i.e., association memories) connected to the lost stimuli's columnar circuits, as well as corresponding frontal action columns tied to the lost stimuli. The exact connections among all columns are only guesswork at this point, but following extinction there is the lack of activation of the mPFC and OFC columns connected to the ventral striatum and limbic structures. The immediate result of loss is that the primary affective state tied to the lost stimulus fails to activate, leaving only the opponent affective process. There will likely be increased generalized metabolic activity (due to this being new learning related to the absent stimulus) around the involved columnar circuits. The generalized activity in the absence of the prior column activation would simply weaken association column activation (i.e., only noise in the absence of the signal). In the case of loss of a previously rewarding stimulus, this would account for any observed metabolic increases in activity in various cortical regions.

Support for distributed cortical effects was provided in two studies on grief [214],[215]. O'Connor et al. [215] compared individuals with complicated grief versus those with uncomplicated grief. Complicated grief is considered present in an individual experiencing excessively prolonged grief which includes recurrent pangs of painful emotions with intense yearning for the loved one. The authors' goal was to elucidate the neural mechanisms associated with both complicated and uncomplicated grief. The independent variables involved a single photograph of the deceased individual versus a stranger and 15 idiosyncratic grief-related words versus 15 neutral words across 60 trials. Composites of each of the photographs and each of the words served as the presented stimuli. The dependent variables involved fMRI measures in regions of interest. There were several areas (i.e., dACC, insula, PAG) activated in both groups related to pictures of the deceased individuals and the grief words. However, the NAc was the only brain area showing relatively greater activity in the complicated grief group in response to the grief-related words. Although initial analysis revealed no difference between groups in response to photographs of the deceased, the authors were sensitive to the likelihood of habituation (i.e., new versus old learning) since the same photograph was used in all trials. They then compared groups on the basis of the first third of the trials only and found results in which complicated grief participants showed greater NAc activation. When the increased NAc activation was observed, it was correlated with self-reported yearning for the deceased. No correlation was found between NAc activity related to length of time since death, participant age, or general positive or negative affect.

The results of the O'Connor et al. [215] study can be used to show the potential explanatory power of a brain-based theoretical model in psychotherapy. I [54] previously suggested that complicated grief is best conceptualized as a traumatic event which leads to a phobic-type response, while the loss aspects follow an opponent-process pattern. In this regard, the situational variables related to the loved one's death represent the trauma and the complicated grief patient finds the activation of the traumatic memory to be emotionally painful. The patient actively avoids situations, thoughts, and conversations which activate the memories of the loss, including many pre-death memories. Conceptually, these traumatic memories are characterized primarily by loss of control without the personal inadequacy/responsibility aspects and are responsive to imaginal exposure procedures. In treatment, the patient goes through two to three detailed descriptions of the events beginning when the patient was notified of the death through the time of returning home after the funeral. Across the one to two-hour session, anxiety peaks quickly with a gradual dissipation. The patient often having improved recollection of details across the descriptions. There is typically a dramatic improvement following the patient engaging in a brief role play at the end of the session which involves saying “good-bye” to the deceased. Once the trauma has been addressed, the patient typically lacks the loss-related depression typical of recent loss. This suggests that despite the complicated grief patient avoiding discussions of the deceased, there is automatic lessening in strength of the opponent-process based upon the absence of the deceased individual in various situations where they were formerly present. Similar complicated grief treatment procedures to those described above have been reported as effective by other authors [216].

The O'Connor et al. [215] study shows that the NAc appears to be involved in response to cortically processed stimuli, both visually and verbally, and associated with perceived yearning. The yearning is certainly one aspect of what patients report during the dysphoria phase of grieving. For normal grief, individuals face the environmental stimuli (thereby activating the theorized associated cortical columns) leading to recollection of the deceased, including the reality of the death, on a frequent basis. Consistent with an opponent-process theory, there is expected gradual deactivation of the associated ventral striatum areas. By avoiding discussions, looking at pictures, or visiting the grave the cortical activation of memories associated with the deceased, complicated grief patients are only successful at controlling exposure to specific stimuli and extended recollection of the loss traumatic experience. In complicated grief subjects, with as few as 30 picture presentations, there was an indication of decreased NAc activation tied to that one specific stimulus. Although such a restricted exposure to a single stimulus likely had little therapeutic effect, it can be used to support the expected alteration in activation of the NAc in response to exposure procedures.

In relation to loss-related depression in pain patients, education is important. The normal process which is typical of natural reactions to significant loss of any kind (e.g., loss of health, loss of job) is discussed and written information is provided. In this case, the depressive symptoms are considered normal and the patient is encouraged to be realistic in facing and accepting permanent losses, with the understanding that the depressive symptoms will gradually dissipate over time.

In relation to a few chronic pain patients, I have effectively treated complicated bereavement in the course of comprehensive care. However, it is only one targeted area related to the neutralization of relevant negative emotional memories. Notably, loss-related depression is frequent with chronic pain patients over the first one to three years after disabling pain develops. That is the time that patients are dealing with multiple losses in areas such as independent functioning, relationships, income, and recreational activities. I have also observed that the healthiest outcome from those losses is an acceptance of one's lack of control in many life situations, but with the associated perception of one's ability to cope with any future losses that may be encountered. Treatment mainly involves education on why the depression is expected to develop (normalizing the experience) and healthy ways to accept and deal with the experience. This is typically done within a constructivist approach directed toward the development of new life meaning.

## 11. Conclusions

The current paper has attempted to explain neurophysiological pain processing based on a cortical columnar theory. Within the discussion, novel views on the role of the basal ganglia, cerebellum, and cingulate cortex have been presented. Additionally, an applied clinical treatment model based on the DSM has been discussed. Three specific areas amenable to psychological treatment were discussed, each of which has been utilized in a clinical setting with chronic pain patients. It is hoped that the information will lead to a consideration of researchers and clinicians to fully evaluate the value of both the DSM and CBM.

## Footnotes

1 An important note is that, although the use of verbal and emotion interpreter identification may sound like modules, the DSM indicates each has multiple independent columnar circuits with specific connections to the lower-order columns involved in behavior production (e.g., premotor to motor cortex). There are also rostral and/or dorsal higher-order columns that can exert control on the interpreters’ circuitry and simultaneous control of other lower-order PFC columns associated with other functions.
